# Comparison of XEN gel stent for management of open-angle glaucoma: a systematic review and meta-analysis

**DOI:** 10.7717/peerj.21133

**Published:** 2026-06-09

**Authors:** Xiangjun Fu, Guoliang Li, Juan He, Hongyi Luo, Ruijue Peng, Yilian Cheng, Jie Peng, Shiyan Chen, Chao Qu

**Affiliations:** 1 School of Medicine, University of Electronic Science and Technology of China, Chengdu, China; 2 Chengdu University of Traditional Chinese Medicine, Chengdu, China; 3 Sichuan Provincial People’s Hospital, Chengdu, China; 4 Sichuan Key Laboratory for Disease Gene Study Chinese Academy of Sciences Sichuan Translational Medicine Research Hospital, Chengdu, Sichuan Province, China

**Keywords:** Open-angle glaucoma, XEN gel stent, Glaucoma surgery, Minimally invasive glaucoma surgery

## Abstract

**Background:**

The comparative efficacy of XEN gel stent implantation, either as a standalone procedure (XEN-only) or combined with phacoemulsification (Phaco-XEN), for the treatment of open-angle glaucoma (OAG) warrants further investigation. This systematic review and meta-analysis evaluated the efficacy of XEN-only and Phaco-XEN procedures compared to trabeculectomy (TB).

**Methods:**

Five major electronic databases, including PubMed, ScienceDirect, and the Cochrane Library, were systematically searched from inception to September 1, 2025. We compared clinical outcomes among XEN-only, Phaco-XEN, and TB groups. Stratification analyses examined outcomes based on follow-up duration and geographic location. Efficacy endpoints included intraocular pressure (IOP), number of anti-glaucoma medications (NOAM), and bleb needling rates. Sensitivity analyses were performed to assess potential biases.

**Results:**

A total of 56 eligible studies were included. (1) Both XEN-only and Phaco-XEN procedures achieved significant IOP reductions from baseline (*P* < 0.001). When comparing XEN-only versus Phaco-XEN, no statistically significant differences were observed in postoperative IOP (MD: −0.22; *P* = 0.06), NOAM (MD: 0.10; *P* = 0.06), or bleb needling rates (RR: 1.79; *P* = 0.06). (2) Compared with trabeculectomy (TB), there was no significant difference in postoperative NOAM for XEN-only (MD: −0.16; *P* = 0.16), but XEN-only had lower postoperative IOP (MD: −0.93; *P* = 0.0003). However, extremely high heterogeneity (*I*^2^ = 86%) suggests this finding should be interpreted with caution. Conversely, XEN-only surgery was associated with a significantly higher rate of bleb needling compared to TB surgery (RR: 3.09; *P* = 0.001). Hyphema and bleb needling were identified as the most frequent postoperative complications.

**Conclusion:**

Both XEN-only and Phaco-XEN surgeries provide effective IOP reduction. There was no statistically significant difference in postoperative NOAM reduction between XEN-only and Phaco-XEN surgeries (*P* = 0.06). TB surgery remains superior regarding lower postoperative bleb needling rates compared to XEN-only surgery.

## Introduction

Glaucoma remains the leading cause of irreversible blindness worldwide. As global life expectancy rises, the prevalence of this condition is projected to increase significantly. Current estimates suggest that the number of individuals affected by glaucoma will reach 111.8 million by 2040, with open-angle glaucoma (OAG) accounting for the majority of cases globally (Buffault et al., 2020; Friedman et al., 2002). While traditional glaucoma filtration surgeries, such as trabeculectomy (TB), remain the “gold standard” for lowering intraocular pressure (IOP) (Costa et al., 1993; Gedde et al., 2021), they are often associated with significant postoperative complications and intensive follow-up requirements. Consequently, Minimally Invasive Glaucoma Surgery (MIGS) has emerged as a therapeutic alternative, aiming to achieve effective IOP control and reduce medication dependence while minimizing tissue trauma and postoperative risk (Kerr, Wang & Barton, 2017).

The XEN Gel Stent (AqueSys, Inc., Aliso Viejo, CA, USA) represents a significant advancement in subconjunctival MIGS devices. It is a 6-mm hydrophilic tube designed to create a permanent channel from the anterior chamber to the subconjunctival space, thereby facilitating aqueous humor outflow (Chao et al., 2021; Scheres et al., 2021). The stent is available in three inner lumen diameters: 45, 63, and 140 µm. Composed of glutaraldehyde-crosslinked gelatin derived from porcine dermis, the implant is designed to ensure biocompatibility and minimize the inflammatory response associated with synthetic materials (Hohberger, Welge-Lüßen & Lämmer, 2018; Urcola & Garay-Aramburu, 2021). Clinically, the XEN stent is indicated for the management of refractory OAG, including primary open-angle glaucoma, pseudoexfoliative glaucoma, and pigmentary glaucoma, particularly in patients where previous surgical treatments have failed or IOP remains uncontrolled despite maximal medical therapy (Fea et al., 2020; Fernández-García et al., 2020b; Hengerer et al., 2018; Hohberger, Welge-Lüßen & Lämmer, 2018; Panarelli et al., 2023).

Recent longitudinal studies indexed in PubMed have begun to elucidate the long-term efficacy of the XEN gel stent, providing durability data extending beyond five years (Lavin-Dapena et al., 2021; Busch et al., 2023; Gan et al., 2024). Concurrently, research has increasingly focused on device-specific complications—such as conjunctival erosion, stent degradation, and late-onset bleb fibrosis—contributing to a more comprehensive understanding of the device’s safety profile in real-world settings (Laroche, Nkrumah & Ng, 2019; Oddone et al., 2021; Schubert, 1996; Brown et al., 2025; Amarasekera, Shankar & Razeghinejad, 2024; Brown et al., 2025; Gan et al., 2024; Zhang et al., 2023; Marchese et al., 2025). Despite this evolving evidence base, consensus regarding the comparative efficacy of XEN implantation *versus* traditional trabeculectomy remains elusive (Panarelli et al., 2023). For instance, while Marcos Parra et al. (2019) reported similar IOP-lowering effects and medication reduction between XEN and TB, (Schlenker et al. (2017) highlighted the superior safety profile of XEN. Conversely, other studies have documented severe adverse events, including permanent vision loss and device-related failure modes like intraluminal obstruction (Gillmann et al., 2018; Lim & Lim, 2018; Pérez-Torregrosa et al., 2016; Widder et al., 2019). Furthermore, the comparative efficacy of standalone XEN implantation (XEN-only) *versus* XEN combined with phacoemulsification (Phaco-XEN) has yielded inconsistent results across different study populations (Lim et al., 2021; Olgun, Aktas & Ucgul, 2020; Zhang, Hirunyachote & Jampel, 2015). To address these discrepancies and guide clinical decision-making, we conducted a systematic review and meta-analysis. This study aims to comprehensively assess the efficacy and safety of XEN gel stent implantation for OAG, specifically comparing outcomes between XEN-only, Phaco-XEN, and traditional TB surgery.

## Methods

### Inclusion and exclusion criteria

The eligibility criteria were defined based on the PICOS (Population, Intervention, Comparison, Outcomes, Study design) framework.

### Inclusion criteria

(1) Adult patients with a confirmed diagnosis of OAG. (2) Studies involving primary open-angle glaucoma (POAG) or secondary OAG. (3) A minimum follow-up duration of 6 months. (4) Studies reporting at least one of the following outcomes: preoperative and postoperative IOP, number of anti-glaucoma medications (NOAM), or bleb needling rates. (5) Full-text articles published in peer-reviewed journals.

### Exclusion criteria

(1) *In vitro* or animal studies. (2) Case reports, editorials, reviews, meta-analyses, or conference abstracts. (3) Studies with a small sample size (*n* < 10). (4) Literature with incomplete data on outcome metrics (pre- and post-surgical IOP, pre- and post-surgical NOAM, and bleb needling rate).

### Search strategy

**Figure 1:**
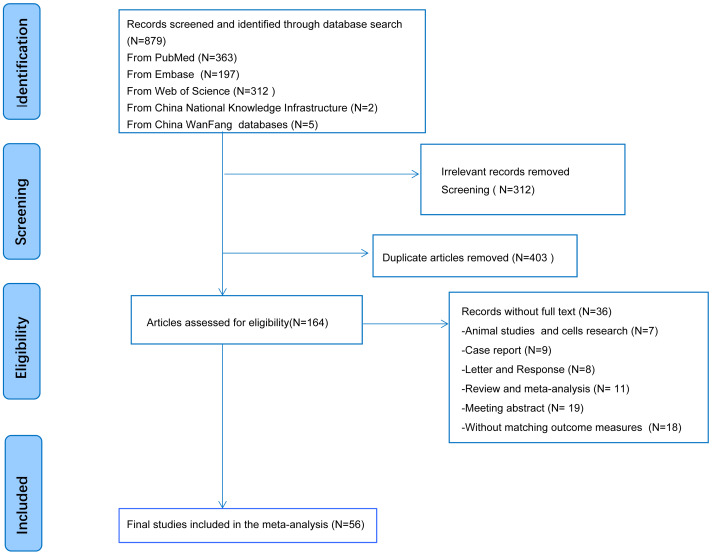
PRISMA flow diagram delineating search, screening, and eligibility assessment process.

This study was performed in accordance with the Preferred Reporting Items for Systematic Reviews and Meta-Analyses (PRISMA) guidelines. The protocol was registered with the Open Science Framework (ID: 10.17605/OSF.IO/5ENHP). A systematic search was conducted in PubMed, EMBASE, Web of Science, Cochrane Library, China National Knowledge Infrastructure (CNKI), and WanFang databases from inception to February 1, 2026. Search terms included combinations of keywords related to “XEN gel stent” (*e.g.*, XEN implant, collagen stent) and “open-angle glaucoma”. The detailed search strategy is provided in Table S1. The screening process is illustrated in the PRISMA flow diagram (Fig. 1).

### Data extraction and quality assessment

Data extraction was performed independently by two investigators (G.L.L. and J.H.) and cross-verified for accuracy. Extracted data included: author, publication year, country, sample size (number of eyes), demographics (age, sex), surgical procedure, and follow-up duration. Corresponding authors were contacted to request missing data when necessary.

Quality assessment: The methodological quality and risk of bias were independently evaluated by two reviewers. The Cochrane Risk of Bias Tool (RoB 2) was used for randomized controlled trials (RCTs), while the Newcastle-Ottawa Scale (NOS) was applied to non-randomized observational studies. Studies with an NOS score < 6 were considered low quality (Sterne et al., 2019; Vu et al., 2021; Munn et al., 2015). Discrepancies during extraction or assessment were resolved through discussion or consultation with a third reviewer (X.J.F.).

### Statistical analysis

Meta-analysis was conducted using Review Manager (RevMan version 5.4; The Cochrane Collaboration). Pooled risk ratios (RR) for dichotomous variables and weighted mean differences (MD) for continuous variables were calculated using the inverse variance method with 95% confidence intervals (CI). Heterogeneity was assessed using Cochran’s Q test (*P* < 0.10 indicating significance) and the I^2^ statistic. A random-effects model was employed when significant heterogeneity was observed (I^2^ > 50%) to provide conservative confidence interval estimates, accounting for between-study variance due to differing surgical techniques and patient characteristics. Conversely, a fixed-effects model was applied when low heterogeneity was observed (I^2^ < 50%).

Funnel plots were generated to assess publication bias. Sensitivity analyses were performed using the leave-one-out method to evaluate the robustness of the results. Subgroup analyses were conducted to explore potential sources of heterogeneity, stratified by follow-up duration and geographic location. A *P*-value < 0.05 was considered statistically significant.

**Definition of Success**: Given the lack of standardized definitions in the literature, composite success criteria were established for this analysis. Complete success was defined as postoperative IOP ≤ 15 mmHg, no medication use, and >20% reduction from baseline. Qualified success was defined as IOP ≤ 15 mmHg, >30% reduction from baseline, with or without medication.

## Results

### Systematic review and study inclusion

Our search strategy initially yielded 879 publications from PubMed (*n* = 363), Embase (*n* = 197), Web of Science (*n* = 312), CNKI (*n* = 2), and Wanfang databases (*n* = 5). Of these, 403 studies were excluded due to duplication. After title and abstract screening, 312 studies were excluded. The abstract sections of 164 potentially relevant full-text studies were carefully reviewed for compliance with our criteria. A final selection process excluded studies that lacked full-text availability (*n* = 36), involved animal or cell research (*n* = 7), were case reports (*n* = 9), comprised letters or responses (*n* = 8), were review or meta-analyses (*n* = 11), constituted meeting abstracts (*n* = 19), or did not include at least one of the outcome metrics (*n* = 18). Finally, 56 trials were included for quantitative analysis. Table 1 summarizes the characteristics of the included literature.

**Table 1:** Main characteristics of the studies included in the meta-analysis.

**First Author**	**Publish Year**	**Country**	**Study design**	**Eyes included**	**Male/ Female**	**Age** **(Mean ± SD)**	**Surgical implantation**	**Follow-up**	**Using or not using MMC**
Sheybani et al. (2015)	2015	US	Prospective	37	14/23	69.6 ± 7.7	phaco-XEN	12 m	NR
Fea et al. (2017)	2017	Italy	Prospective	12	5/6	71.3 ± 10	XEN	12 m	0.02% MMC
Pérez-Torregrosa et al. (2016)	2016	Spain	Prospective	30	5/13	76 ± 5.85	phaco-XEN	12 m	0,01% MMC, 1% ACh
Galal et al. (2017)	2017	Germany	Prospective	13	6/4	73.1 ± 10	phaco-XEN	12 m	0.01% MMC
Olate-Pérez et al. (2017)	2017	Spain	Prospective	30	5/13	76 ± 5.85	phaco-XEN	12 m	NR
De Gregorio et al. (2018)	2018	Italy	Prospective	41	13/20	74 ± 7.1	phaco-XEN	12 m	0.1 mL MMC diluted to 0.2 mg/mL
Hengerer et al. (2018)	2018	Germany	Retrospective	110	23/32	69.6 ± 13.7	XEN	12 m	0.1 ml MMC
Karimi et al. (2018)	2018	UK	Retrospective	17	9/8	76.1	XEN	12 m	0.1 mL of 0.2 mg/mL MMC
Tan, Walkden & Au (2018)	2018	UK	Retrospective	43	18/21	70.1 ± 13.8	XEN	12 m	0.2 mg/ml MMC, 0.1ml 50 mg/ml 5-FU
Widder et al. (2018)	2018	Germany	Retrospective	261	92/141	73 ± 11	XEN; phaco-XEN	18 m	0.1 mL MMC diluted to 0.1 mg/ mL
Hengerer, Auffarth & Conrad-Hengerer (2019)	2019	Germany	Retrospective	148	89/59	68.4 ± 13.9	XEN	12 m	NR
Kalina, Kalina & Brown (2019)	2019	USA	Prospective	47	14/28	78.15 ± 8.55	XEN; phaco-XEN	12 m	0.1 ml MMC 0.2 mg/ml 0.1ml 5-FU
Lenzhofer et al. (2019a)	2019	Austria	Prospective	64	35/29	NR	XEN	48 m	NR
Gillmann et al. (2021)	2020	Switzerland	Prospective	37	10/27	77.7 ± 9.1	XEN	24 m	0.02% 0.1 ml MMC
Fernández-García et al. (2020a)	2020	Spain	Retrospective	40	17/23	77.31 ± 6.33	XEN	36 m	0.1 mlMMC diluted to 0.02 mg/ml
Fernández-García et al. (2020b)	2020	Spain	Retrospective	93	22/41	74 ± 8	XEN	36 m	NR
Teus et al. (2019)	2019	Spain	Retrospective	48	27/21	72.7 ± 12.51	XEN	48 m	0.2 mg/ml mmc, 0.1ml50 mg/ml 5-FU
Post et al. (2020)	2020	Poland	Prospective	20	6/11	69.85 ± 4.69	XEN	12 m	0.1 ml 0.02% MMC
Tan et al. (2021)	2021	US	Retrospective	50	NR	71.0 ± 13.4	XEN	12 m	MMC 5FU
Schargus et al. (2020)	2020	Germany	Retrospective	113	73/80	70.2 ± 10.8	XEN	12 m	0.01% MMC 0.1 ml, 5-FU (5 mg/0.1 ml)
Scheres et al. (2021)	2020	Netherlands	Retrospective	82	41/41	69 ± 8	XEN	24 m	0.1 ml 5-FU diluted to 50 mg/ml fx1 0.1 ml MMC diluted to 0.2 mg/ml
Lenzhofer et al. (2019b)	2019	Austria	Prospective	137	67/70	75.2 ± 7.0	XEN; phaco-XEN	24 m	0.05–0.1 ml, 4–8 μ g MMC
Lenzhofer et al. (2019c)	2019	Austria	Prospective	66	28/38	72.2 ± 12.5	XEN; phaco-XEN	12 m	0.05–0.1 ml, 4–8 lg MMC
Mansouri et al. (2019)	2019	Switzerland	Prospective	149	32/81	74.4 ± 9.4	XEN; phaco-XEN	24 m	0.1 ml dose of 0.02% MMC., dexamethasone
Marcos Parra et al. (2019)	2019	Spain	Retrospective	121	59/62	71.2 ± 11.7	XEN; phaco-XEN;TB	12 m	0.1 ml dose of 0.01% MMC, dexamethasone 0.1%
Midha et al. (2019)	2019	Switzerland	Prospective	149	63/70	74.4 ± 9.6	XEN; phaco-XEN	24 m	0.01% MMC
Reitsamer et al. (2022)	2019	Austria	Prospective	161	90/95	71.8 ± 10.5	XEN; phaco-XEN	24 m	(97.5%) of the eyes used MMC; 19 (9.4%) and 22 (10.8%) eyes used steroids or VEGF
Barão et al. (2020)	2020	Portugal	Retrospective	42	12/30	71.7 ± 12	XEN; phaco-XEN	18 m	0.1 mL MMC diluted to 0.2 mg/mL; 20 μ g
Buffault et al. (2020)	2020	France	Retrospective	107	58/49	68.3 ± 10.8	XEN; phaco-XEN	6 m	0.1 ml of MMC
Gillmann et al. (2020)	2020	Switzerland	Prospective	92	23/45	76.3 ± 9.1	XEN; phaco-XEN	36 m	0.1 mL of MMC 0.02%
Ibáñez Muñoz et al. (2020)	2020	Spain	Retrospective	73	39/34	79.7 ± 8.2	XEN; phaco-XEN	12 m	0.1 mL MMC diluted to 0.2 mg/mL; 20 μ g
Laborda-Guirao, Cubero-Parra & Hidalgo-Torres (2020)	2020	Spain	Retrospective	80	42/38	74.0 ± 10.4	XEN; phaco-XEN	12 m	0.1 mL of MMC 0.02%
Midha et al. (2020)	2020	Switzerland	Prospective	51	15/36	74.4 ± 9.4	XEN; phaco-XEN	24 m	NR
Olgun, Aktas & Ucgul (2020)	2020	Turkey	Retrospective	221	42/72	65.8 ± 10.6	XEN; phaco-XEN; TB	24 m	NR
Olgun et al. (2021)	2020	Turkey	Retrospective	80	29/35	61.1 ± 12.1	XEN; TB	3 m	0.1 mL of MMC diluted to 0.2 mg/mL.
Wałek et al. (2020)	2020	Poland	Prospective	39	19/20	67	XEN; phaco-XEN	24 m	MMC, 5-fu
Chao et al. (2021)	2021	China	Retrospective	37	24/14	53.4 ± 13.6	XEN	12 m	NR
Oddone et al. (2021)	2021	Italy	Prospective	108	84/84	69.1 ± 12.9	XEN; phaco-XEN	6 m	NR
Reitsamer et al. (2019)	2021	Austria	Retrospective	212	83/94	76 ± 7.1	XEN; phaco-XEN	36 m	MMC /VEGF
Urcola & Garay-Aramburu (2021)	2021	Spain	Retrospective	20	3/7	76.1 ± 12	XEN; phaco-XEN	12 m	0.1 m% MMC 0.01 ml
Cappelli et al. (2022)	2020	Spain	Prospective	11	2/9	78.8	XEN; phaco-XEN	18 m	MMC
Fea et al. (2020)	2020	Italy	Prospective	298	149/149	70.3 ± 11.8	XEN; phaco-XEN	12 m	0.1 mL MMC 0.02%
Rather, Vold & Mcfarland (2020)	2020	USA	Retrospective	92	31/35	75.3	XEN; phaco-XEN	12 m	MMC 0.2 mg/mL
Hohberger, Welge-Lüßen & Lämmer (2018)	2018	Germany	Retrospective	111	64/47	68 ± 14	XEN	6 m	0.01% MMC, 10 μ g
Laroche, Nkrumah & Ng (2019)	2019	US	Retrospective	12	NR	NR	XEN	12 m	NR
Cappelli et al. (2022)	2021	Italy	Retrospective	68	43/25	73.5 ± 13.4	XEN; TB	36 m	0.12 mL MMC diluted to 0.2 mg/mL
Subaşıet al. (2021)	2021	Turkey	Retrospective	30	17/13	66.17 ± 8.19	phaco-XEN	24 m	0.1 mL MMC 0.02%
Theilig et al. (2020)	2020	Germany	Retrospective	200	108/92	70.6	XEN; phaco-XEN, TB	12 m	MMC diluted to 0.2 mg/Ml, 5-FU
Mansouri et al. (2018)	2018	Swiss	Prospective	139	81/58	74.4 ± 9.4	XEN; phaco-XEN	12 m	0.1 mL MMC 0.02%
Teus et al. (2019)	2019	Spain	Cross sectional	25	18/7	71.45 ± 11.06	XEN; TB	35 m	0.02%MMC
Papazoglou et al. (2024)	2024	Swiss	Retrospective	345	230/115	73.6 ± 12.5	XEN; phaco-XEN	24 m	NR
Arnould et al. (2024)	2024	Austria	Retrospective	646	258/257	72.4. ± 11.1	XEN; phaco-XEN	24 m	0.05 mL, 0.2–0.4 mg/mL
Rauchegger, T (Pirani et al., 2024)	2024	Austria	Retrospective	142	56/86	74.9 ± 9.4	XEN; TB	36 m	0.028 mg/ml
Busch, T (Jin et al., 2025)	2023	Swiss	Retrospective	119	48/55	70.6 ± 12.1	XEN, phaco-XEN	48 m	0.1 mL MMC 0.2 mg/mL
Pirani, V (Gan et al., 2024a)	2024	Italy	Retrospective	31	20/11	65.6 ± 12.3	XEN	6 m	0.1 mg/ mL MMC
Torbey Julien[]	2023	Swiss	Prospective	170	89/37	78.1 ± 9.2	XEN, phaco-XEN	60 m	NR

**Notes.**

Abbreviations PHACO phacoemulsification TB trabeculectomy n number of eyes SD standard deviation CI confidence interval NOAM number of antiglaucoma medications MMC mitomycin C NR not reported ACh acetylchol

The meta-analysis included 5,079 patients (5,720 eyes). Sample sizes ranged from 10 to 298 eyes, with participant ages between 40 and 88 years. Study designs included prospective (*n* = 23), retrospective (*n* = 32), and cross-sectional cohorts (*n* = 1). Geographically, the majority originated from Europe (*n* = 47), followed by North America (*n* = 5), Asia (*n* = 4), and Oceania (*n* = 1). The comparisons analyzed were: XEN-only (*n* = 18), Phaco-XEN alone (*n* = 6), XEN-only *vs.* Phaco-XEN (*n* = 25), XEN-only *vs.* TB (*n* = 5), and three-arm comparisons (*n* = 3). Follow-up durations ranged from 3 to 60 months.

### Efficacy of XEN-only surgery

#### Changes in IOP before and after XEN-only surgery

**Figure 2:**
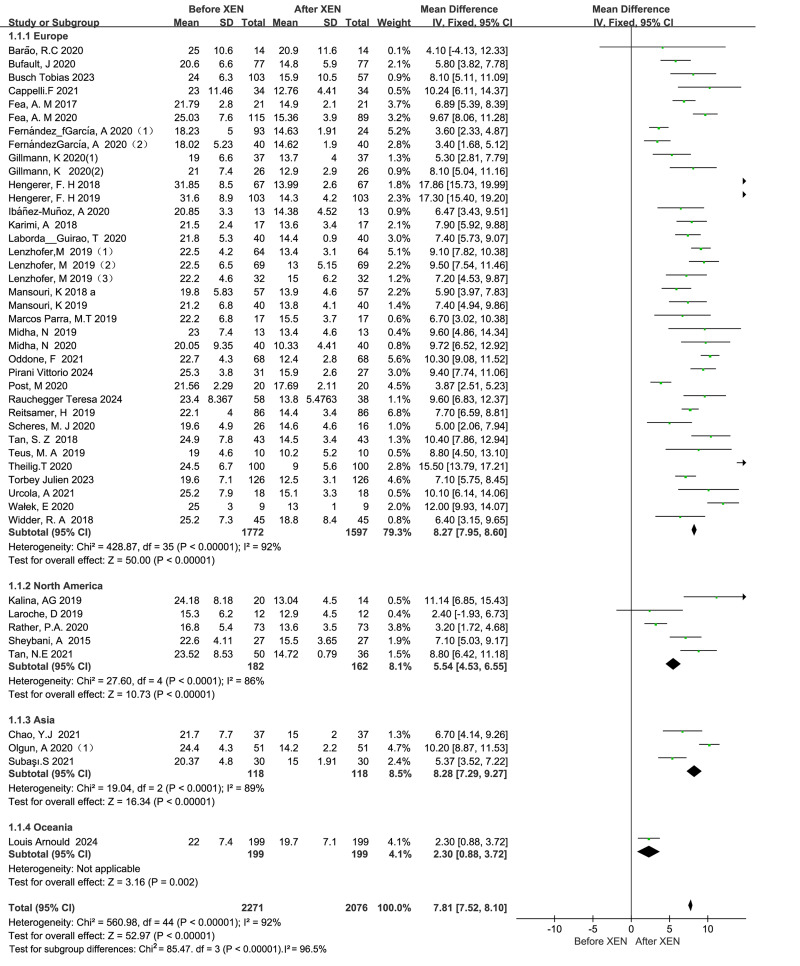
Forest plot of comparison of IOP before and after XEN-only surgery (stratified by geographic).

Analysis of 45 studies (2,271 preoperative *vs.* 2,076 postoperative eyes) demonstrated a significant reduction in IOP following XEN-only implantation. Using a random-effects model (I^2^ >50%), the pooled analysis showed a substantial IOP decrease (MD: 7.81, 95% CI [7.52–8.10], *P* < 0.001). Subgroup analysis by geography confirmed significant IOP reductions across all regions: Europe (MD: 8.27, *P* < 0.001), Asia (MD: 8.28, *P* < 0.001), North America (MD: 5.54, *P* < 0.001), and Oceania (MD: 2.30, *P* = 0.002) (Fig. 2).

#### IOP changes associated with XEN-only surgery across different follow-up periods

**Figure 3:**
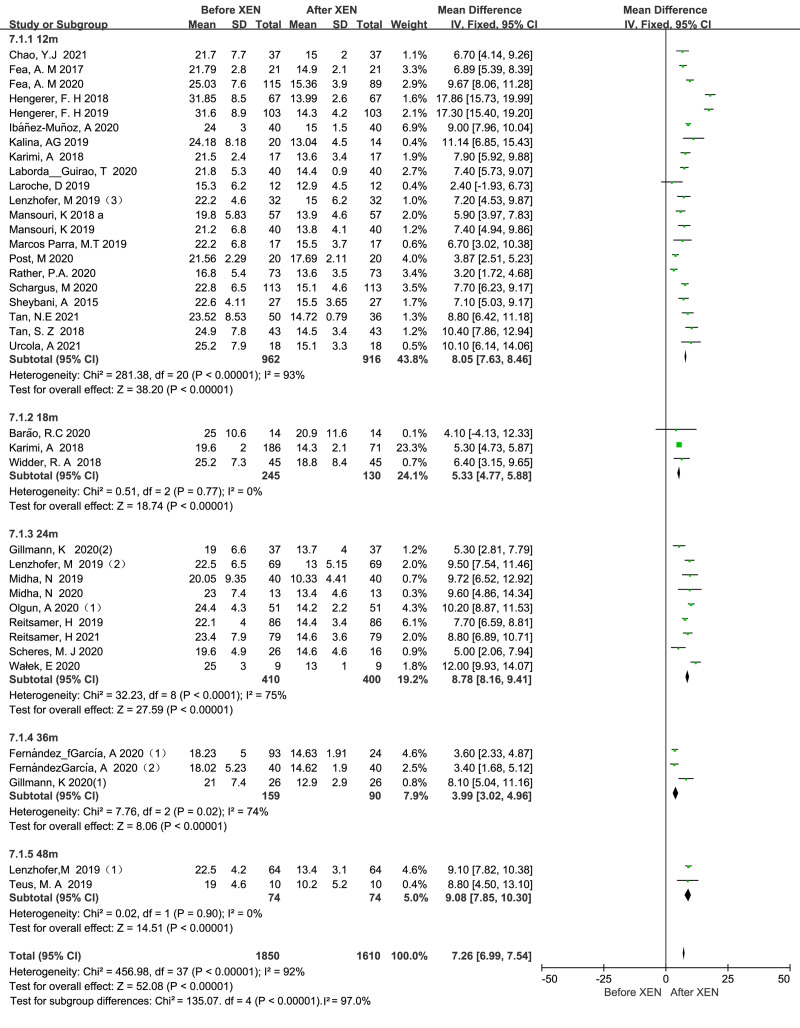
Forest plot of comparing IOP according to follow-up time before and after XEN-only surgery (stratified by time).

Postoperative IOP was significantly lower than preoperative levels across all time points (Fig. 3). The analysis was stratified into five follow-up periods: 12 months (*n* = 21), 18 months (*n* = 3), 24 months (*n* = 9), 36 months (*n* = 3), and 48 months (*n* = 2). The preoperative IOP was lower than postoperative IOP after XEN-only surgery at 12 months (MD: 8.05, *P* < 0.001), 18 months (MD: 5.33, *P* < 0.001), 24 months (MD: 8.78, *P* < 0.001), 36 months (MD: 3.99, *P* < 0.001), and 48 months (MD: 9.08, *P* < 0.001). Significant reductions persisted from 12 months through 48 months, indicating sustained long-term efficacy.

#### IOP changes after XEN surgery: short-term and long-term follow-up periods

A pooled analysis of 41 studies demonstrated a significant reduction in IOP after XEN surgery. When stratified by follow-up duration, both short-term (≤ 12 months; 24 studies) and long-term (12–36 months; 17 studies) periods showed markedly lower postoperative IOP compared to preoperative levels (short-term: MD = 8.89, *P* < 0.001; long-term: MD = 6.81, *P* < 0.001). The overall analysis confirmed a substantial IOP decrease after XEN surgery (MD: 8.04, 95% CI [6.84–9.24], *P* < 0.001) (Fig. S5).

### Efficacy of phaco-XEN surgery

#### Changes in IOP before and after phaco-XEN surgery

Phaco-XEN surgery resulted in significant IOP reduction compared to baseline (MD: 6.73, 95% CI [6.43–7.02], *P* < 0.001). Subgroup analysis confirmed efficacy across Europe (MD: 6.39, *P* < 0.001), North America (MD: 6.00, *P* < 0.001), and Asia (MD: 11.40, *P* < 0.001) (Fig. S1).

#### IOP changes before and after phaco-XEN surgery based on the follow-up time

All time intervals demonstrated significant reductions in IOP following phaco-XEN surgery compared to preoperative levels: 6-month (MD: 6.76, *P* < 0.001), 12-month (MD: 6.27, *P* < 0.001), 18-month (MD: 4.32, *P* < 0.001), 24-month (MD: 8.01, *P* < 0.001), and 36-month follow-up (MD: 7.1, *P* < 0.001). Significant IOP reductions were maintained at all intervals from 6 months to 36 months (Fig. S2).

#### IOP changes after phaco-XEN surgery: short-term and long-term follow-up

In the phaco-XEN cohort (27 studies), postoperative IOP was significantly reduced across both short-term (≤ 12 months; 17 studies) and long-term (12–36 months; 10 studies) follow-up intervals (short-term: MD = 7.35, *P* < 0.001; long-term: MD = 6.74, *P* < 0.001). The overall effect also indicated a pronounced IOP reduction compared to preoperative values (MD: 7.10, 95% CI [5.97–8.23], *P* < 0.001) (Fig. S6).

### Comparison of the efficacy of XEN-only and phaco-XEN

#### Postoperative IOP follow-up period for XEN-only and phaco-XEN procedures

**Figure 4:**
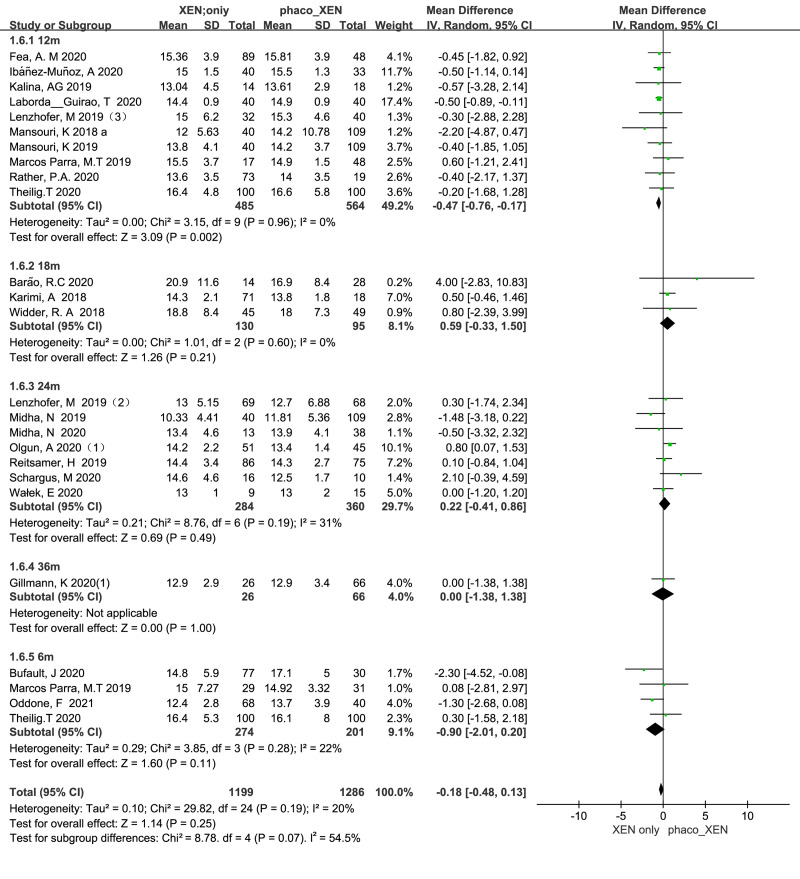
Forest plot of comparing IOP at follow-up after XEN**-**only *versus* phaco-XEN (stratified by time).

There was no statistically significant difference in overall postoperative IOP between XEN-only and Phaco-XEN groups (MD: −0.22, 95% CI [−0.46–0.01], *P* = 0.06) (Fig. 4). Subgroup analysis revealed that XEN-only achieved significantly lower IOP at 12 months compared to Phaco-XEN (MD: −0.47; *P* = 0.002), but no significant differences were observed at 6 months (MD: −0.93, *P* = 0.05), 18 months (MD: 0.59, *P* = 0.21), 24 months (MD: 0.32, *P* = 0.18), or 36 months (MD: 0.00, *P* = 1.00).

#### XEN-only *vs* phaco-XEN surgery for the treatment of postoperative NOAM

**Figure 5:**
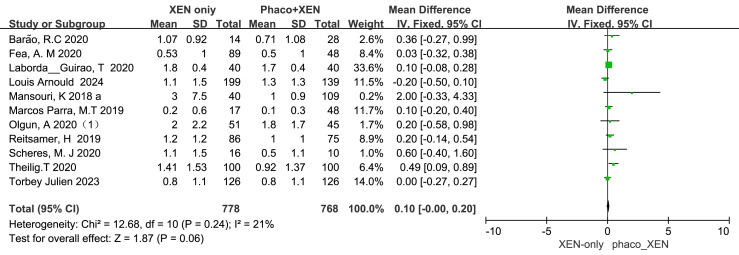
Forest plot of comparing postoperative NOAM between XEN-only and phaco-XEN surgerys.

This analysis compared the postoperative number of anti-glaucoma medications (NOAM) between patients who underwent XEN-only surgery (*n* = 778 eyes) and those who underwent phaco-XEN surgery (*n* = 768 eyes). There was no statistically significant difference in postoperative NOAM between the two groups (MD: 0.10, 95% CI [0.00–0.20], *P* = 0.06) (Fig. 5).

#### Bleb needling rate after XEN-only *vs.* phaco-XEN surgery

**Figure 6:**
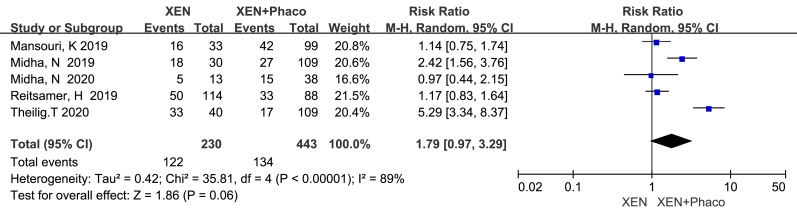
Forest plot of comparing the bleb needling rate after the XEN-only and phaco-XEN surgery.

Five studies compared the postoperative bleb needling rate in patients who underwent phaco-XEN (*n* = 230 eyes) and XEN-only (*n* = 443 eyes) surgeries. There was no statistically significant difference in the bleb needling rate between the XEN-only and phaco-XEN groups (RR: 1.79, 95% CI [0.97–3.29], I^2^ = 89%, *P* = 0.06) (Fig. 6).

### Comparison of the efficacy of XEN-only *vs.* TB surgery

#### Preoperative and postoperative IOP comparing XEN-only *vs.* TB surgery

##### Preoperative IOP.

**Figure 7:**
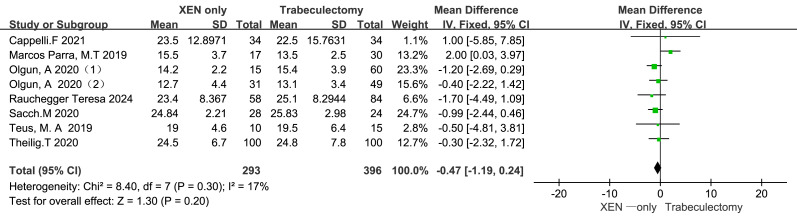
Forest plot of comparing preoperative IOP between XEN-only and TB surgery.

Eight studies compared the preoperative IOP between patients who underwent XEN-only (*n* = 293 eyes) and TB (*n* = 396 eyes) surgery. Following heterogeneity assessment, a fixed-effects model was employed for data synthesis (MD: −0.47, I^2^ = 17%, *P* = 0.2). There was no significant difference between the two groups in terms of preoperative IOP, suggesting comparable baseline IOP levels between treatment groups (Fig. 7).

##### Postoperative IOP.

**Figure 8:**
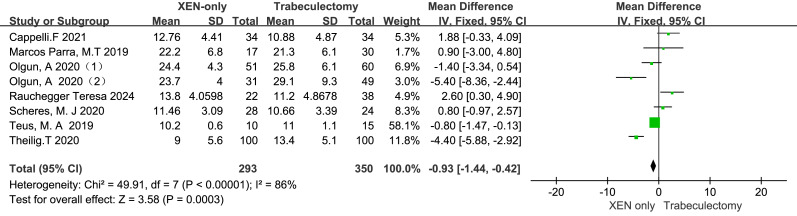
Forest plot of comparing postoperative IOP between XEN-only and TB surgery.

Eight studies were included in this analysis comparing postoperative IOP between XEN-only (*n* = 293 eyes) and TB surgery (*n* = 350 eyes). Significant heterogeneity was observed (I^2^ = 86%), necessitating the use of a random-effects model for data synthesis. The pooled analysis revealed that XEN-only has better efficacy in reducing IOP after surgery compared to TB procedures (MD: −0.93, *P* = 0.0003) (Fig. 8).

#### NOAM before and after XEN-only *vs* TB surgery

##### Preoperative NOAM.

**Figure 9:**
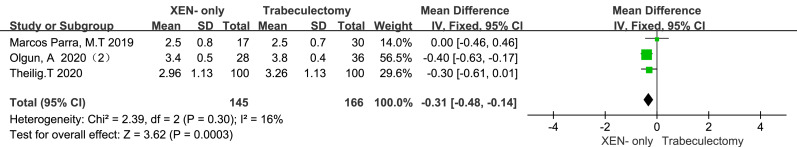
Forest plot of comparing preoperative NOAM of XEN-only and TB surgery.

Three studies comparing preoperative NOAM between XEN-only (*n* = 145 eyes) and TB (*n* = 166 eyes) groups were pooled. Given low heterogeneity (I^2^ = 16%), a fixed-effects model was applied. The meta-analysis showed that preoperative NOAM was significantly lower in the XEN-only group than in the TB group (MD: −0.31, 95% CI [−0.48 to −0.14], *P* = 0.0003) (Fig. 9).

##### Postoperative NOAM.

**Figure 10:**
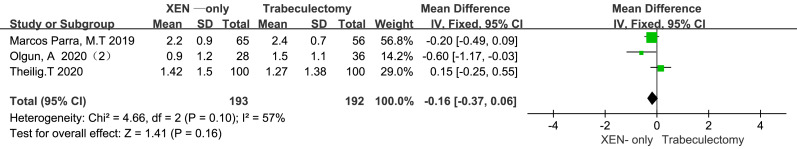
Forest plot of comparing postoperative NOAM of XEN-only and TB surgery.

Three studies reporting postoperative NOAM in XEN-only (*n* = 192 eyes) and TB surgery (*n* = 193 eyes) groups were synthesized. Moderate heterogeneity was observed (I^2^ = 57%), warranting a random-effects model. The pooled results indicated no statistically significant difference in NOAM reduction between the two procedures (MD: −0.16, 95% CI [−0.37–0.06], *P* = 0.16) (Fig. 10).

#### Bleb needling rate after XEN-only *vs.* TB surgery

**Figure 11:**
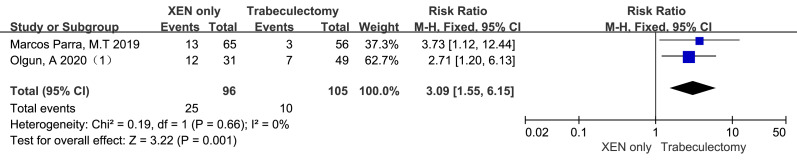
Forest plot of comparing the bleb needling rate of XEN-only group and TB group after surgery.

Two studies compared the bleb needling rate between patients who underwent XEN-only surgery (*n* = 96 eyes) and TB surgery (*n* = 105 eyes). Heterogeneity testing revealed moderate variability (I^2^ = 57%), necessitating the use of a random-effects model for data synthesis. The pooled analysis demonstrated that the postoperative bleb needling rate of TB surgery is lower than that of XEN-only surgery (RR: 3.09, 95% CI [1.55–6.15], *P* = 0.001) (Fig. 11).

### Surgical complications

Hyphema and bleb needling were the two most common adverse events after XEN gel stent implantation surgery. The XEN gel stent group had a significantly lower incidence of adverse events, including hyphema compared to the control group (*P* < 0.05). Adverse events occurring in the XEN gel stent group and the control group in the controlled clinical trial are shown in Table 2.

**Table 2:** Comparison of complications between XEN-only group and control group 2.

**Adverse event**	**NO. of studies**	**Crude rate n/N (%)**		**RR** ** (95% CI)**	**Test for heterogeneity**			**Test for overall effect**	
		**XEN-only**	**Control**		**X** ^**2**^	**I** ^**2**^	**P**	**Z**	**P**
Hyphema	5	45/413	63/389	1.04 [0.79, 1.37]	8.88	5	0.06	2.18	0.03
Hypotony	3	41/172	34/213	1.62 [0.97, 2.71]	2.31	14	0.31	1.83	0.07
Choroidal detachment	2	20/134	11/134	1.96 [0.90, 4.26]	0.57	0	0.49	1.69	0.09
Choroidaleffusion	2	60/72	83/112	4.02 [0.56, 28.72]	0.01	0	0.45	1.39	0.17
Needling	4	50/237	74/268	0.8 [0.49, 1.33]	13.16	77	0.004	0.86	0.39
AC flattening	3	61/137	95/168	0.39 [0.15, 1.06]	7.51	73	0.02	1.84	0.07

**Notes.**

Abbreviations AC anterior chamber RR Relative Risk 95% CI 95% confidence interval

The control group consisted of phaco-XEN and TB.

### Comparison of complete surgical success rates

#### Postoperative complete success rates of XEN-only *vs* phaco-XEN

A pooled analysis of 14 studies (XEN-only: *n* = 922 eyes; phaco-XEN: *n* = 960 eyes) indicated a significantly higher complete success rate for the XEN-only group compared to the phaco-XEN group (RR: 1.5, 95% CI [1.22–1.85], *P* = 0.0001). Data were synthesized using a random-effects model due to considerable heterogeneity (I^2^ > 50%) (Fig. S3).

#### Postoperative complete success rate of XEN-only *vs* TB

Three studies (XEN-only: *n* = 231 eyes; TB: *n* = 237 eyes) were pooled using a fixed-effects model given low heterogeneity (I^2^ < 50%). The meta-analysis showed a significantly lower complete success rate for XEN-only surgery compared to TB (RR: 0.65, 95% CI [0.45–0.95], *P* = 0.03) (Fig. S3).

#### Comparison of surgical complete success rate at different follow-up times

##### Comparison of postoperative complete success rate of XEN-only *vs.* phaco-XEN at 12 months of follow-up.

Three studies comparing XEN-only (*n* = 193 eyes) and phaco-XEN (*n* = 252 eyes) groups at 12 months found no statistically significant difference in complete success rates (RR: 1.44, 95% CI [0.93–2.21], *P* = 0.10). Data were pooled using a random-effects model (I^2^ > 50%) (Fig. S4).

##### Comparison of the postoperative complete success rate of XEN-only *vs.* phaco-XEN at 24 months of follow-up.

Similarly, two studies comparing XEN-only (*n* = 153 eyes) and phaco-XEN (*n* = 143 eyes) groups at 24 months showed no significant difference in complete success rates (RR: 1.08, 95% CI [0.65–1.81], *P* = 0.77). A random-effects model was used to account for considerable heterogeneity (I^2^ > 50%) (Fig. S4).

### Sensitivity analysis and publication bias

Sensitivity analyses using the leave-one-out method confirmed the robustness of the results. Funnel plots indicated no evidence of significant publication bias (Figs. S7–S22).

## Discussion

This systematic review synthesized data from 56 clinical studies selected from an initial pool of 879 records. This updated meta-analysis provides a comprehensive quantitative evaluation of XEN implantation for OAG, assessing efficacy outcomes including IOP, NOAM, bleb needling rates, and surgical success. By analyzing comparative efficacy and safety profiles across XEN-only, Phaco-XEN, and TB groups, our study aims to clarify the role of XEN implantation in current glaucoma management and address inconsistencies observed in previous research.

### Efficacy after XEN implantation

Consistent with earlier literature (Lenzhofer et al., 2019c; Post et al., 2020; Reitsamer et al., 2019), data on the long-term effectiveness of XEN gel stents beyond one year remains relatively limited. However, recent studies published in 2024 and 2025 have begun to shed light on 2- to 3-year outcomes (Widder et al., 2019; Papazoglou et al., 2024; Torbey et al., 2023), providing crucial evidence regarding the long-term sustainability of XEN implantation (Arriola-Villalobos et al., 2012; Busch et al., 2023; Pirani et al., 2024; Gan et al., 2024). Our analysis confirms that XEN implantation demonstrates robust efficacy for OAG treatment. Both XEN-only and Phaco-XEN procedures yielded significant reductions in IOP and medication burden postoperatively compared to baseline.

Despite stratification by geographic location, significant heterogeneity persisted in outcomes across studies from Asia, North America, and Europe. This variability suggests the influence of unmeasured factors, such as differences in disease severity, patient baseline characteristics, racial physiological differences (Fan et al., 2019; Martin et al., 1985; Wilson et al., 1985), or adjunctive antimetabolite protocols, necessitating further investigation to disentangle these sources of heterogeneity. In terms of surgical outcomes, the highest reported complete success rate reached 68.8%, with qualified success rates up to 90.6%. Notably, some cohorts achieved complete medication independence post-surgery, highlighting the potential of XEN implantation to eliminate the medication burden—a benefit often less consistent in other MIGS procedures (Friedman et al., 2002; Panarelli et al., 2023; Schlenker et al., 2017; Widder et al., 2018). Subgroup analyses stratified by follow-up duration further confirmed that XEN-only surgery maintains a sustained IOP reduction from 12 to 48 months postoperatively.

### Comparison of XEN-only *vs.* Phaco-XEN

Consensus regarding the comparative IOP-lowering efficacy of XEN-only *versus* Phaco-XEN remains elusive. While Mansouri et al. (2018) observed a greater IOP reduction with combined surgery (56.1% *vs.* 20%), other investigators, including Barão et al. (2020) and Rather, Vold & Mcfarland (2020), reported superior outcomes in the XEN-only group (Mansouri et al., 2019). Conversely, Reitsamer et al. (2022) and others (Ibáñez Muñoz et al., 2020; Post et al., 2020) found no significant difference between the two approaches. In our meta-analysis, no statistically significant difference was found in postoperative IOP reduction between XEN-only and Phaco-XEN (*P* = 0.06). While the *P*-value was borderline, strictly speaking, the null hypothesis could not be rejected.

When evaluating secondary outcomes, previous studies have suggested potential differences in medication burden and needling requirements (Gillmann et al., 2021; Reitsamer et al., 2022). However, regarding safety and medication burden, our study found no statistically significant difference in NOAM or bleb needling rates between the two groups (*P* = 0.06 for both). Given that these findings did not reach the threshold for statistical significance, we refrain from claiming superiority for either procedure; instead, the results suggest comparable safety and medication outcomes based on current evidence.

Recent studies by Mansouri et al. (2018) and Marcos Parra et al. (2019) suggest that standalone XEN implantation may offer superior short-term IOP reduction compared to combined surgery. This observation may be attributed to the pro-inflammatory effect of phacoemulsification, which could potentially compromise bleb function and elevate postoperative IOP in combined procedures (Hansen et al., 1995; Mansouri et al., 2019; Zhang, Hirunyachote & Jampel, 2015). Furthermore, the substantial heterogeneity observed in our meta-analysis may also stem from variations in surgical techniques, such as the placement of the stent (superior *vs.* nasal), the extent of conjunctival dissection, and the variable concentrations of Mitomycin C (MMC) used (Sheybani, Reitsamer & Ahmed, 2015; Suñer et al., 1997; Brown et al., 2025).

Regarding potential confounders, some researchers argue that prior ocular history, such as intraocular lens implantation, may interfere with XEN-only outcomes (Poley et al., 2009). However, Buffault et al. (2020) and Widder et al. (2018) did not find significant differences in IOP or medication requirements when comparing patients with prior surgical history to those without, suggesting that comprehensive preoperative evaluation and precise stent placement are likely the critical determinants of outcomes.

### Comparison of XEN-only *vs* trabeculectomy

The comparative efficacy of XEN-only *versus* TB remains a subject of debate in the literature. Some studies have reported comparable IOP reductions between XEN implantation alone and TB, with no statistically significant differences observed (Jung et al., 2014; Kuroda et al., 2001; Marcos Parra et al., 2019; Murthy et al., 2006; Reitsamer et al., 2022; Song et al., 2016). For instance, Karimi et al. (2018) found that IOP decreased by 46% within 2 years after XEN implantation alone, comparable to the 48% decrease typically seen with TB at 1 year. Conversely, long-term data at 36 months have shown that the TB group maintained lower mean IOP values with less fluctuation compared to the gel stent group (Cappelli et al., 2022; Wagner et al., 2020). In our current meta-analysis, the pooled data indicated that XEN-only yielded a statistically greater IOP reduction compared to TB surgery (MD: −0.93 mmHg; *P* < 0.001), a finding consistent with some expert opinions (Nikita & Murdoch, 2018; Widder et al., 2020). However, this statistical superiority must be interpreted with caution due to the substantial heterogeneity (I^2^ = 86%) observed in our analysis. It is possible that patient selection bias in retrospective studies favored XEN, where cases with less severe glaucoma were selected for stenting, while more complex cases underwent TB. Therefore, we conclude that XEN demonstrates efficacy that is at least comparable to TB, but definitive superiority regarding IOP reduction remains to be confirmed by large-scale RCTs. Theoretically, the shared aqueous outflow pathways, combined with the minimally invasive nature of XEN, may contribute to these favorable dynamics (Panarelli et al., 2023).

Regarding postoperative management, consistent with previous literature (Kirwan et al., 2013; Schlenker et al., 2017), our analysis confirms that the bleb needling rate in TB surgery is significantly lower than in XEN-only implantation. The variation in results may be attributed to the fact that previous studies mostly analyzed all types of glaucoma, while our study only focused on open-angle glaucoma. Currently, there is still controversy regarding the need for postoperative bleb needling, and it should be considered a complication (Tsai et al., 2015). Beyond the focus on open-angle glaucoma in our study, the observed variations in bleb needling rates could also stem from disparities in surgical techniques, adjunctive antimetabolite use (*e.g.*, MMC concentration and application method), differing postoperative management protocols, and patient-specific factors such as individual healing responses or previous surgical history. Addressing these factors more consistently in future studies may help clarify the true incidence and management of bleb needling (Brown et al., 2025). 5-FU is widely thought to be an alternative to bleb needling. Further investigation is essential to determine whether conjunctival opening and scar tissue removal are more beneficial outcomes than bleb needling, or whether postoperative injection of 5-FU can improve the success rate (Wałek et al., 2020).

### Surgical success rates

Despite the lack of standardized definitions for surgical success (complete *vs.* qualified) across the literature, we conducted a quantitative synthesis of available data (Widder et al., 2018). Previously, Wagner et al. (2020) and others (Buffault et al., 2020) found similar failure risks between XEN and TB at 12 months. These findings can be attributed to the higher surgical success stability guaranteed by TB surgery and fewer long-term follow-up visits. Regarding complete success rates, our study concludes that phaco-XEN surgery yields lower rates than XEN-only surgery, which in turn has lower rates than TB surgery. As the “gold standard” of filtration surgery, TB surgery achieves stable and superior postoperative success rates. We next compared the complete success rates of XEN-only and phaco-XEN at 12 and 24 months, demonstrating no significant difference between the two groups.

### The role of mitomycin C

The long-term effects of subconjunctival injection of mitomycin C (MMC) and the need for its continued use are currently unknown (Nikita & Murdoch, 2018). The use of MMC during surgery may increase the risk of complications associated with late blisters (Sheybani et al., 2015). In our current analysis, forty-seven studies included MMC, though with inconsistent concentrations and doses (Table 1). 0.02% MMC used by Gabbay et al.’s (2020) may have yielded greater efficacy than the 0.01% concentration generally used. Conversely, Widder et al.’s (2018) technique, involving a sponge with conjunctival stripping, produced more than twice the incidence of bleb needling. The lack of MMC use in the study by Lenzhofer et al. (2019b) may have contributed to the high rate of bleb needling in their study.

### Safety and complications

Safety profiles reported in the literature vary, but XEN generally demonstrates a favorable safety profile compared to TB. Schlenker et al. (2017) reported a lower complication rate in the XEN group (9.7%) compared to TB (16%). This difference is likely attributable to the minimally invasive ab interno approach, sparing of the conjunctiva, and avoidance of iridectomy (Olgun et al., 2021; Novak-Lauš et al., 2019; Jin et al., 2025). Regarding corneal health, XEN-only implantation typically results in minimal endothelial cell loss (ECL). However, combined Phaco-XEN procedures are associated with higher ECL (*e.g.*, 10% *vs.* 2.1%), primarily due to the trauma associated with phacoemulsification (Rotchford & King, 2008). The structural composition is more similar to healthy conjunctival tissue, resulting in less damage to the body (Teus et al., 2019). However, some prospective, non-randomized, multicenter studies have reported a very low incidence of complications in the first year after XEN gel stent implantation (De Gregorio et al., 2018), with a failure rate comparable to other filtration surgeries (Lenzhofer et al., 2019a), and no significant difference, consistent with the results of our study.

Thirteen studies documented postoperative complications in our study. The significant complications observed included hyphema and hypotony, consistent with previous research results (Buffault et al., 2020; Costa et al., 1993; Marcos Parra et al., 2019; Schubert, 1996; Suñer et al., 1997). Some studies have reported that postoperative bleeding and temporary hypotony can resolve spontaneously. The conclusion of this study aligns with the literature, indicating a low overall incidence of complications with XEN implants, which are typically mild and transient, without posing a threat to vision (Buffault et al., 2020; Siriwardena et al., 2000).

Our study indicated that both short-term (12 months) and long-term (12–36 months) postoperative IOP levels are lower than preoperative levels, demonstrating a consistent surgical effect in reducing IOP over both periods. By analyzing IOP changes at different postoperative time points, it can be inferred that XEN gel microstent implantation surgery exhibits corresponding early and long-term effects in controlling IOP in patients with open-angle glaucoma, as well as in terms of medication control. Current studies primarily focus on short-term efficacy within 12 months, while data on the safety and stability of long-term efficacy remain insufficient (Novak-Lauš et al., 2019; Arriola-Villalobos et al., 2012; Wu et al., 2024). The durability of efficacy is a key consideration. Multiple studies have shown that factors such as age, gender, underlying diseases, family history, glaucoma staging, and whether combined surgery is performed may affect the maintenance of efficacy (Rauchegger et al., 2024).

Future perspectives: Current evidence is heavily weighted toward short-term efficacy. Future research should prioritize multi-center longitudinal studies exceeding 10 years, focusing on: (1) ethnic variations in conjunctival healing; (2) the correlation between material degradation and IOP fluctuation; and (3) standardization of long-term follow-up protocols using digital monitoring platforms (Rauchegger et al., 2024; Busch et al., 2023).

### Limitations

Several limitations warrant mention. First, the predominance of European cohorts and the paucity of data on Asian populations introduce potential selection bias and limit generalizability. Second, the inclusion of retrospective studies inherently introduces selection and recall biases. Third, significant heterogeneity observed in outcomes like needling rates suggests that variations in postoperative management and definitions of “success” across institutions affect the precision of pooled estimates. Finally, variability in stent models (predominantly XEN45, with isolated reports of XEN63/140) and baseline comorbidities (*e.g.*, diabetes, hypertension, cardiovascular disease) represents a potential source of confounding that could not be fully adjusted for.

##  Supplemental Information

10.7717/peerj.21133/supp-1 Supplemental Information 1 Supplementary Table

10.7717/peerj.21133/supp-2 Supplemental Information 2 Forest plot of comparing IOP before and after phaco-XEN surgery (stratified by geographic)

10.7717/peerj.21133/supp-3 Supplemental Information 3 Forest plot of Comparison of IOP before and after phaco-XEN surgery (stratified by time)

10.7717/peerj.21133/supp-4 Supplemental Information 4 Forest plot of comparing complete success rate success rate after XEN-onlygroup versus control group

10.7717/peerj.21133/supp-5 Supplemental Information 5 Forest plot of Comparison of postoperative complete success rate success rates of XEN-only vs phaco-XEN at 12 months and 24 months of follow-up

10.7717/peerj.21133/supp-6 Supplemental Information 6 Forest plot of Comparison of IOP before and after XEN-only surgery (stratified by Long term or short-term follow-up time )

10.7717/peerj.21133/supp-7 Supplemental Information 7 Forest plot of Comparison of IOP before and after phaco-XEN surgery (stratified by Long term or short-term follow-up time)

10.7717/peerj.21133/supp-8 Supplemental Information 8 Filled funnel plot of comparing IOP before and after XEN-only surgery (stratified by geographic)

10.7717/peerj.21133/supp-9 Supplemental Information 9 Filled funnel plot of comparing IOP according to follow-up time before and after XEN-only surgery (stratified by time)

10.7717/peerj.21133/supp-10 Supplemental Information 10 Filled funnel plot of comparing IOP at follow-up after XEN-only versus phaco-XEN (stratified by time)

10.7717/peerj.21133/supp-11 Supplemental Information 11 Filled funnel plot of comparing IOP before and after phaco-XEN surgery, stratified by geographic location: The underlying data is fully presented in Supplementary Figure 1

10.7717/peerj.21133/supp-12 Supplemental Information 12 Filled funnel plot of comparing the bleb needling rate after the XEN-only and phaco-XEN surgery

10.7717/peerj.21133/supp-13 Supplemental Information 13 Filled funnel plot of comparing preoperative IOP between XEN-only and TB surgery

10.7717/peerj.21133/supp-14 Supplemental Information 14 Filled funnel plot of comparing postoperative IOP between XEN-only and TB surgery

10.7717/peerj.21133/supp-15 Supplemental Information 15 Filled funnel plot of comparing preoperative NOAM of XEN-only and TB surgery

10.7717/peerj.21133/supp-16 Supplemental Information 16 Filled funnel plot of comparing postoperative NOAM of XEN-only and TB surgery

10.7717/peerj.21133/supp-17 Supplemental Information 17 Filled funnel plot of comparing the bleb needling rate of XEN-only group and TB group after surgery

10.7717/peerj.21133/supp-18 Supplemental Information 18 Filled funnel plot of comparing IOP at follow-up after XEN-only versus phaco-XEN stratified by time: The underlying data is fully presented in Figure 4

10.7717/peerj.21133/supp-19 Supplemental Information 19 Filled funnel plot of comparing postoperative NOAM between XEN-only and phaco-XEN surgerys: The underlying data is fully presented in Figure 5

10.7717/peerj.21133/supp-20 Supplemental Information 20 Filled funnel plot of of comparing complete success rate success rate after XEN-only group versus control group

10.7717/peerj.21133/supp-21 Supplemental Information 21 Filled funnel plot of Comparison of postoperative complete success rate success rates of XEN-only vs phaco-XEN at 12months and 24 months of follow-up

10.7717/peerj.21133/supp-22 Supplemental Information 22 Filled funnel plot of Comparison of IOP before and after XEN-only surgery (stratified by Long term or short-term follow-up time)

10.7717/peerj.21133/supp-23 Supplemental Information 23 Filled funnel plot of Comparison of IOP before and after phaco-XEN surgery (stratified by Long term or short-term follow-up time)

10.7717/peerj.21133/supp-24 Supplemental Information 24 Risk of bias summary: review authors’ judgements about each risk of bias item for each included study

10.7717/peerj.21133/supp-25 Supplemental Information 25 Risk of bias graph: review authors’ judgements about each risk of bias item presented as percentages across all included studies

10.7717/peerj.21133/supp-26 Supplemental Information 26 PRISMA checklist

10.7717/peerj.21133/supp-27 Supplemental Information 27 Raw data

## Additional Information and Declarations

### Data Availability

The following information was supplied regarding data availability: This is a Systematic Review/Meta-analysis.
