# Supplementary material for: Comparison of XEN gel stent for management of open-angle glaucoma: a systematic review and meta-analysis
Source: PeerJ. 2026 Jun 9;14:e21133. doi: 10.7717/peerj.21133 (PMC13262562; doi:10.7717/peerj.21133)
Supplement: Supplemental Information 1 [file peerj-14-21133-s001.doc]

Supplementary Table I.  Search strategy uesd in each datebase

| Database | Search query | Results |
| --- | --- | --- |
| PubMed | ((open-angle glaucoma[Title/Abstract]) OR (open-angle glaucoma[MeSH Terms])) AND (XEN implant [Title/Abstract]) OR XEN implant [Title/Abstract]) OR Xen Gel Stent [Title/Abstract] OR XEN45 [Title/Abstract] OR XEN-45 [Title/Abstract] OR XEN63 [Title/Abstract] OR XEN-63 [Title/Abstract] OR XEN140 [Title/Abstract] OR XEN-140 [Title/Abstract] OR XEN gel implant [Title/Abstract] OR gel-stent [Title/Abstract] OR gel stent [Title/Abstract] OR gelatin stent [Title/Abstract] OR gelatin-stent [Title/Abstract] OR gelatin implant[Title/Abstract]) | 303 |
| Embase | ('open-angle glaucoma’/exp OR 'open-angle glaucoma':ab,ti) AND ('Xen Gel Stent':ab,ti OR 'Xen implant':ab,ti OR 'XEN45':ab,ti OR 'XEN-45':ab,ti OR 'XEN63':ab,ti OR 'XEN-63':ab,ti OR 'XEN-140':ab,ti OR 'XEN140':ab,tiOR' XEN gel implant':ab,ti OR 'gel-stent':ab,ti OR 'gel stent ':ab,ti OR 'gelatin stent':ab,ti OR 'gelatin-stent':ab,ti OR 'gelatin implant':ab,ti) | 197 |
| Web of Science | TS=“open-angle glaucoma” AND (TS=“Xen Gel Stent” OR TS=“Xen implant” OR TS=“XEN45” OR TS=“XEN-45” OR TS=“XEN gel implant” OR TS=“gel-stent” OR TS=“XEN-63” OR TS=“XEN-140” OR TS=“gel stent” OR TS=“gelatin stent” OR TS=“gelatin-stent” OR TS=“gelatin implant”) | 350 |
| Wanfang | theme:(open-angle glaucoma) * theme:( XEN gel stent) | 5 |
| China National Knowledge Infrastructure (CNKI) | SU=open-angle glaucoma'' AND SU=' XEN gel stent’ | 2 |
