# Supplementary figures and images for: Comparison of XEN gel stent for management of open-angle glaucoma: a systematic review and meta-analysis
Source: PeerJ. 2026 Jun 9;14:e21133. doi: 10.7717/peerj.21133 (PMC13262562; doi:10.7717/peerj.21133)

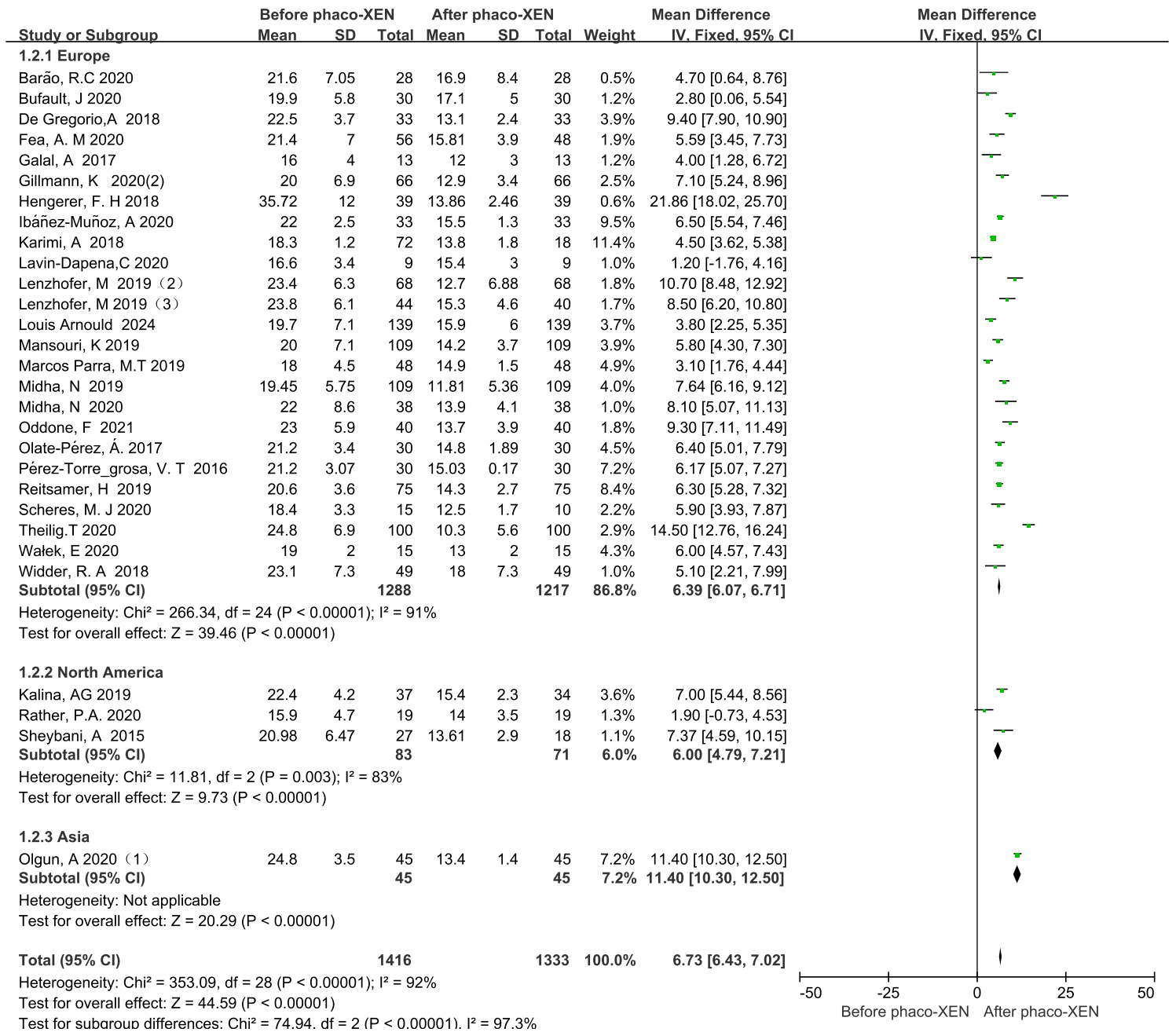

Supplement: Supplemental Information 2 [file peerj-14-21133-s002.pdf]

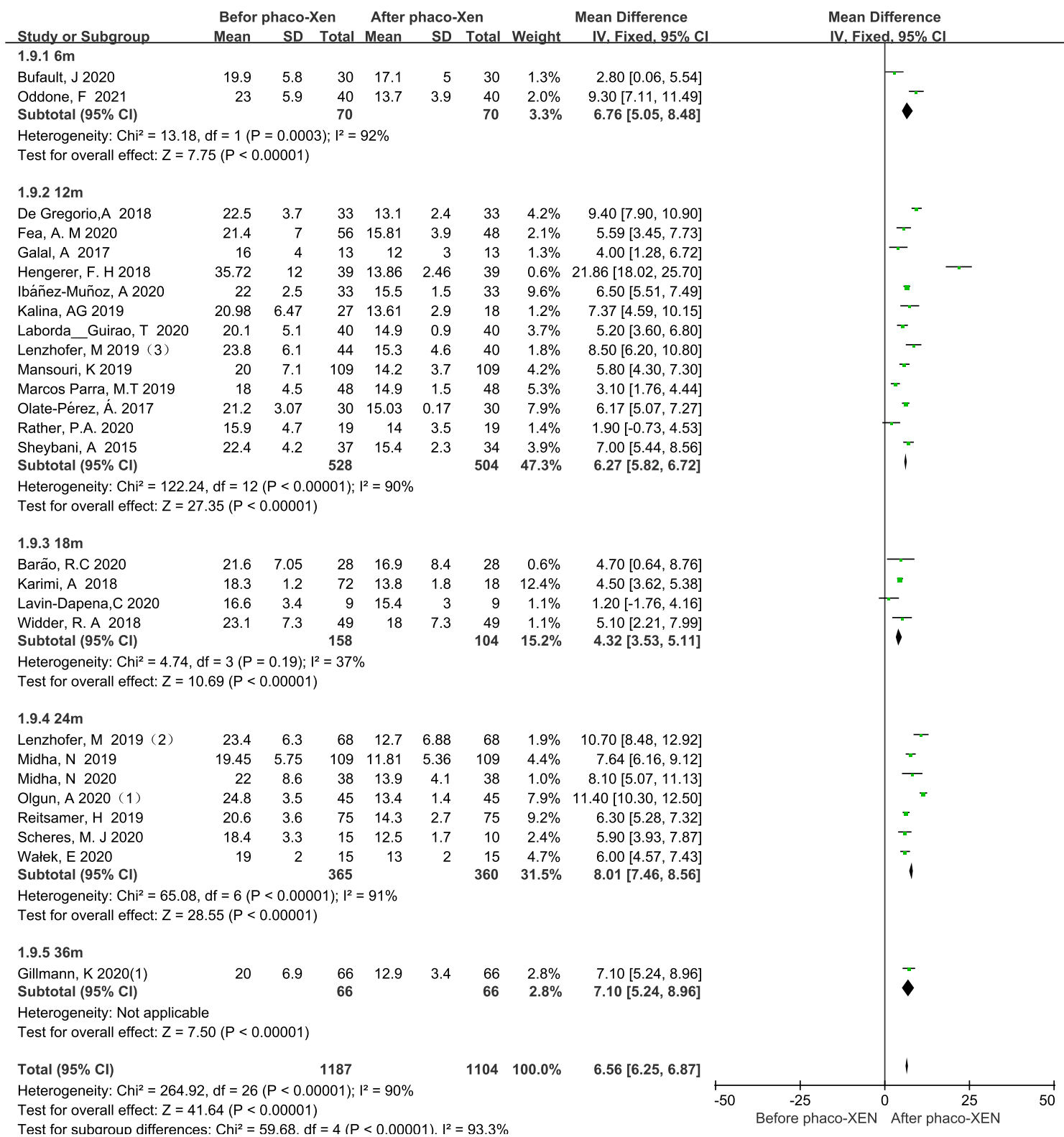

Supplement: Supplemental Information 3 [file peerj-14-21133-s003.pdf]

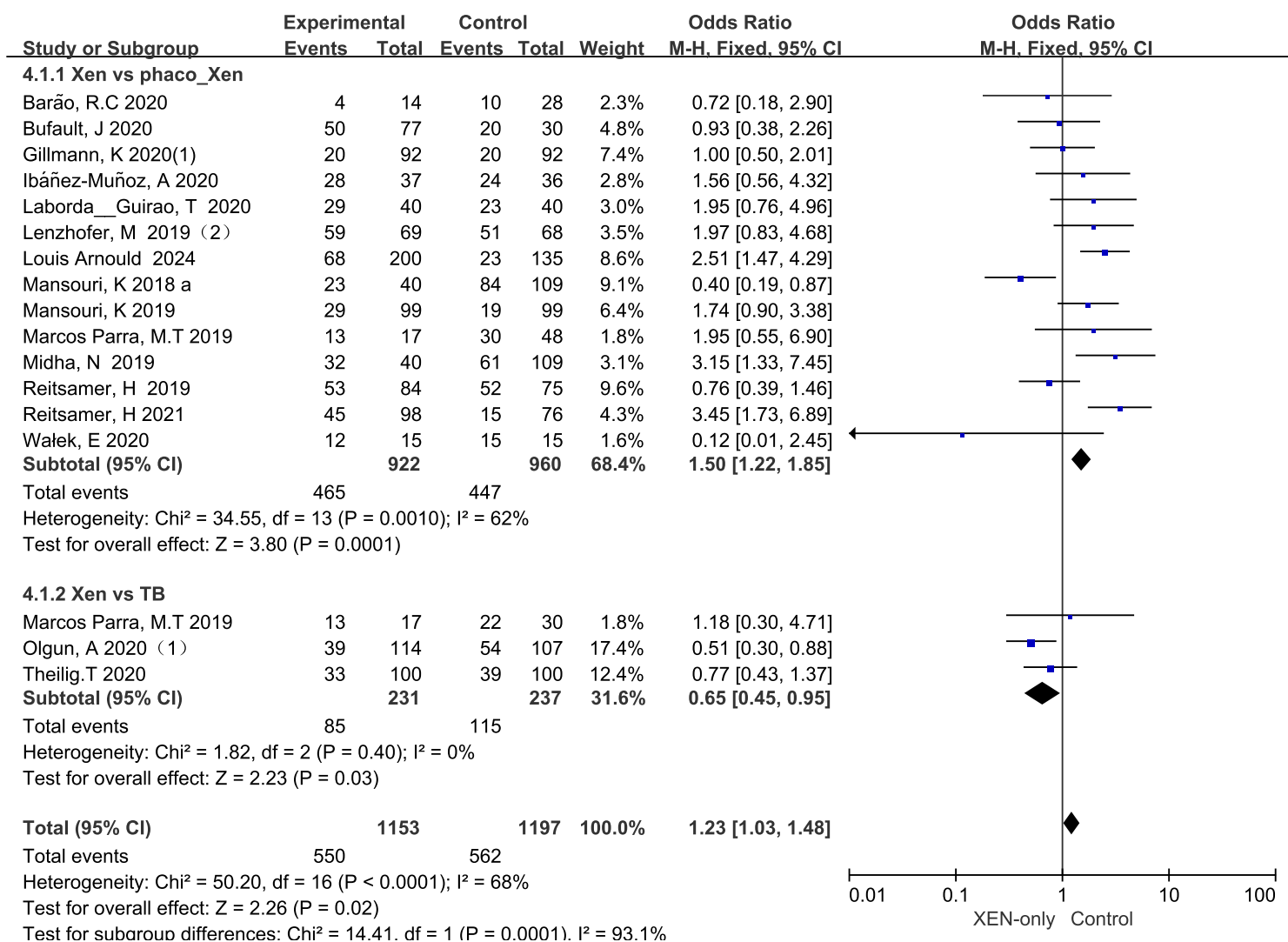

Supplement: Supplemental Information 4 [file peerj-14-21133-s004.pdf]

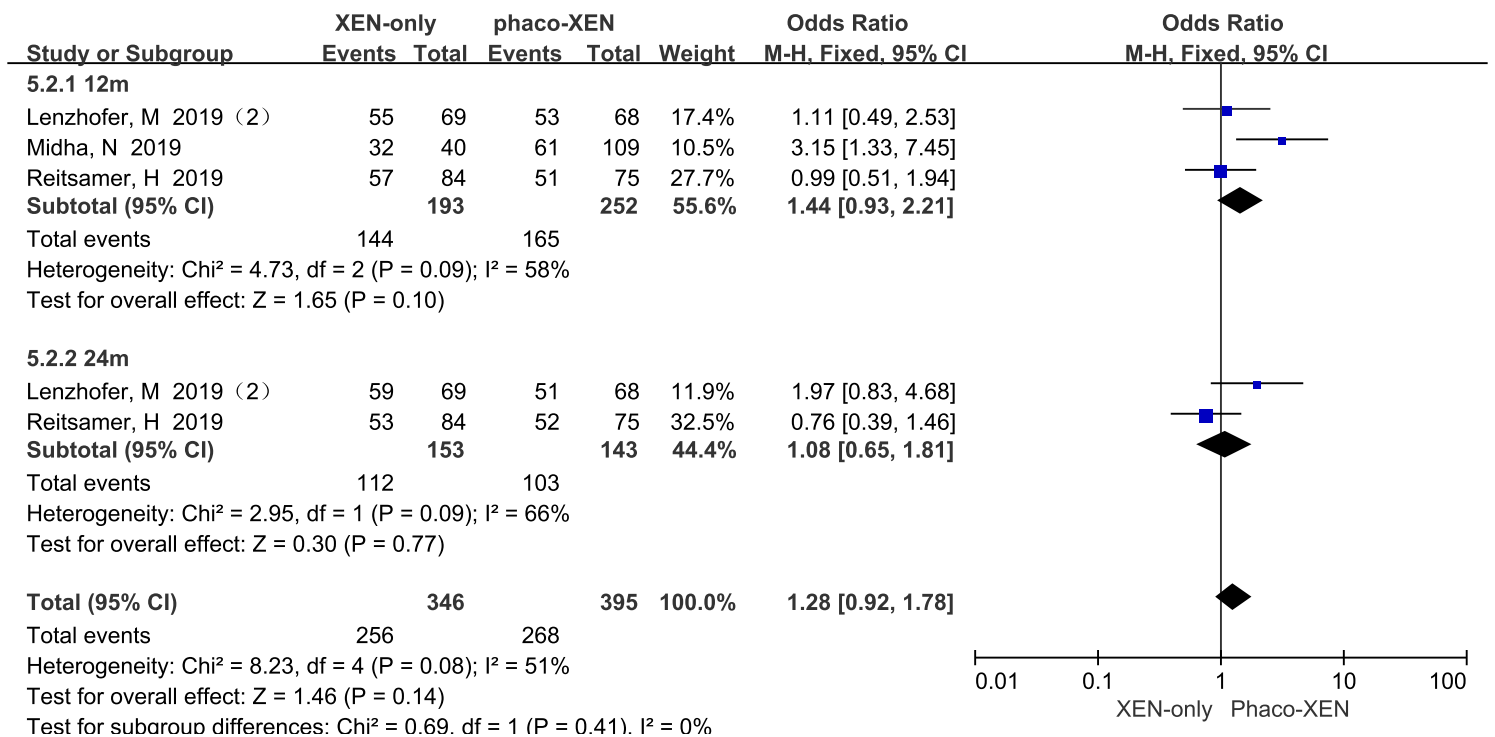

Supplement: Supplemental Information 5 [file peerj-14-21133-s005.pdf]

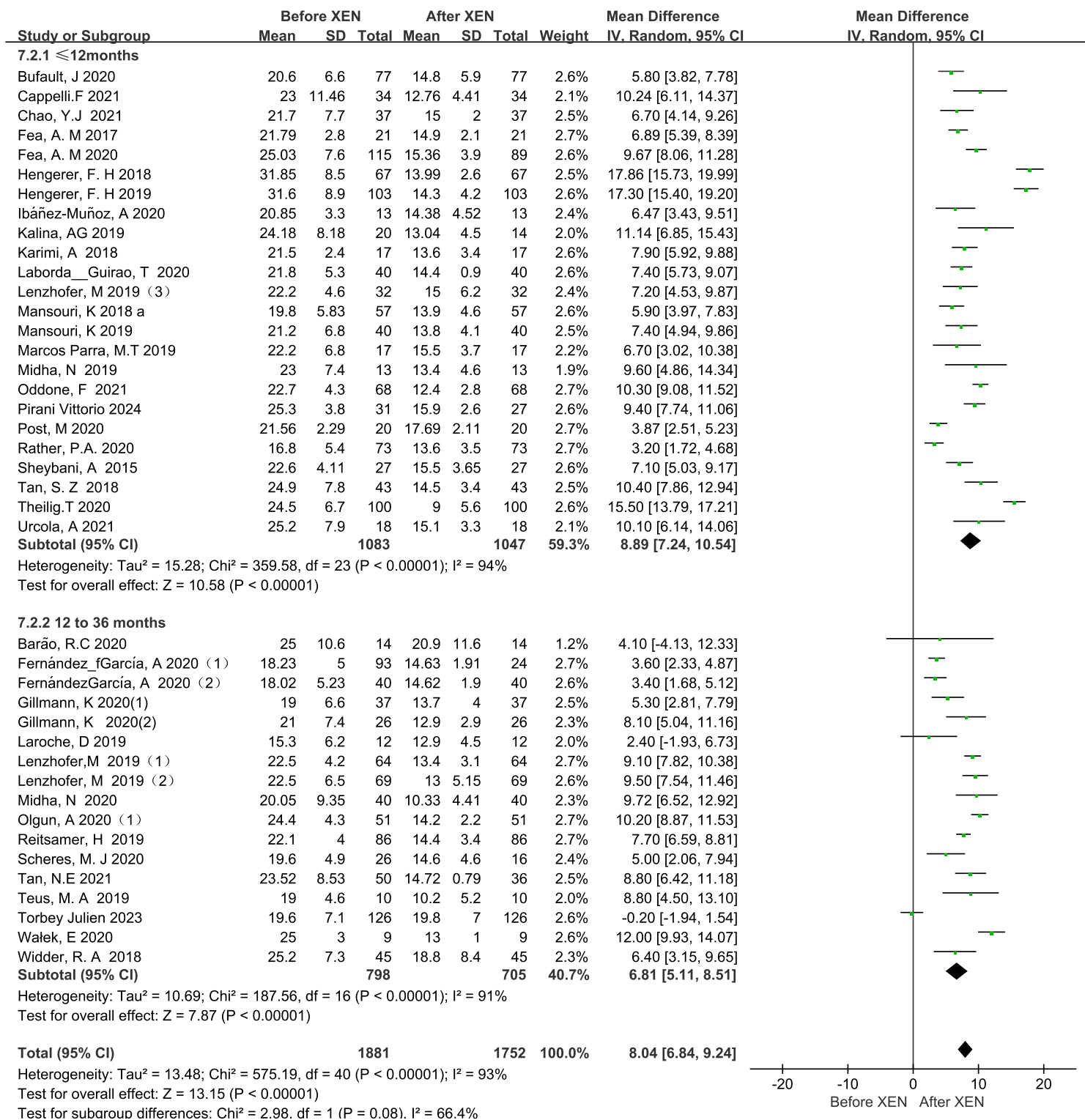

Supplement: Supplemental Information 6 [file peerj-14-21133-s006.pdf]

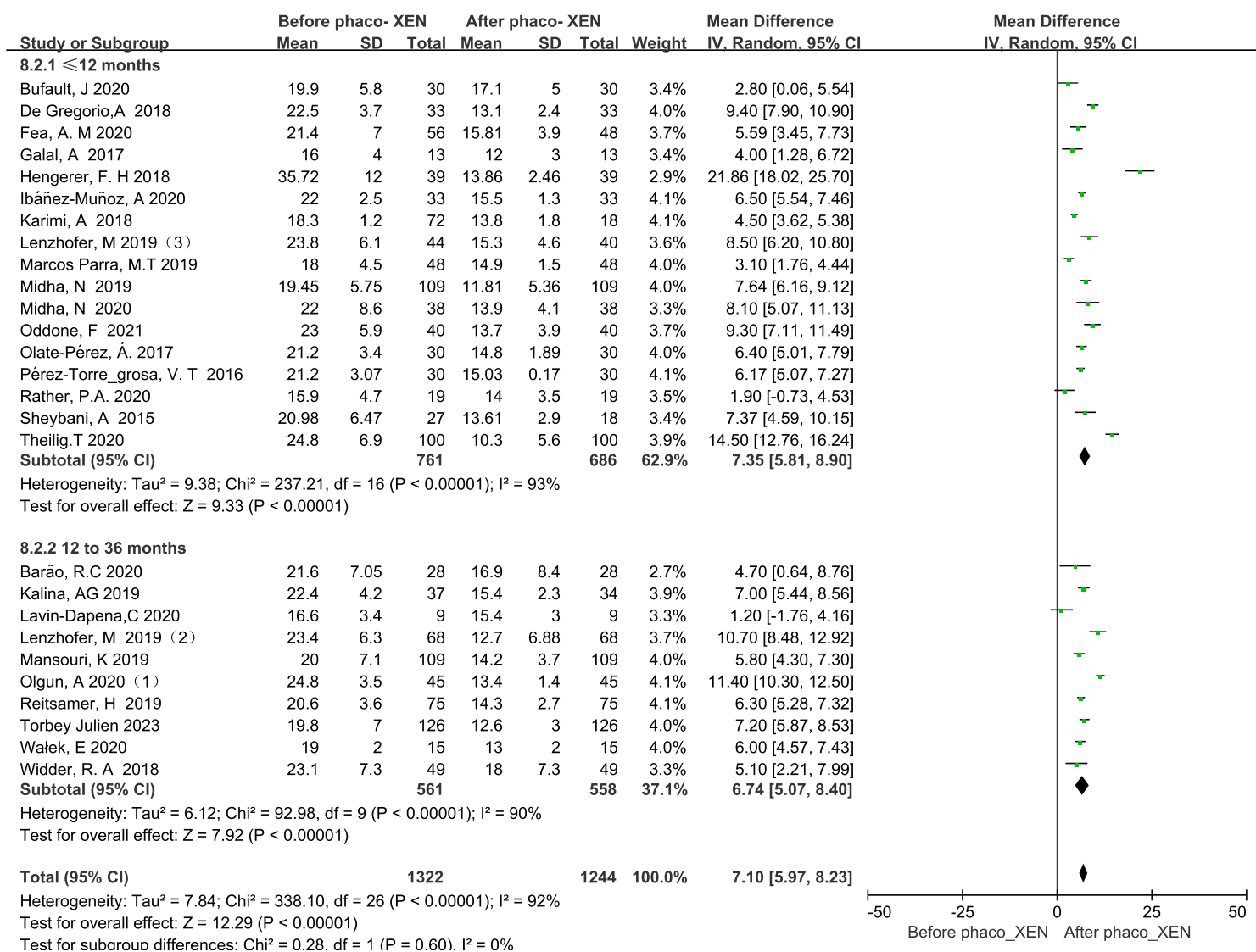

Supplement: Supplemental Information 7 [file peerj-14-21133-s007.pdf]

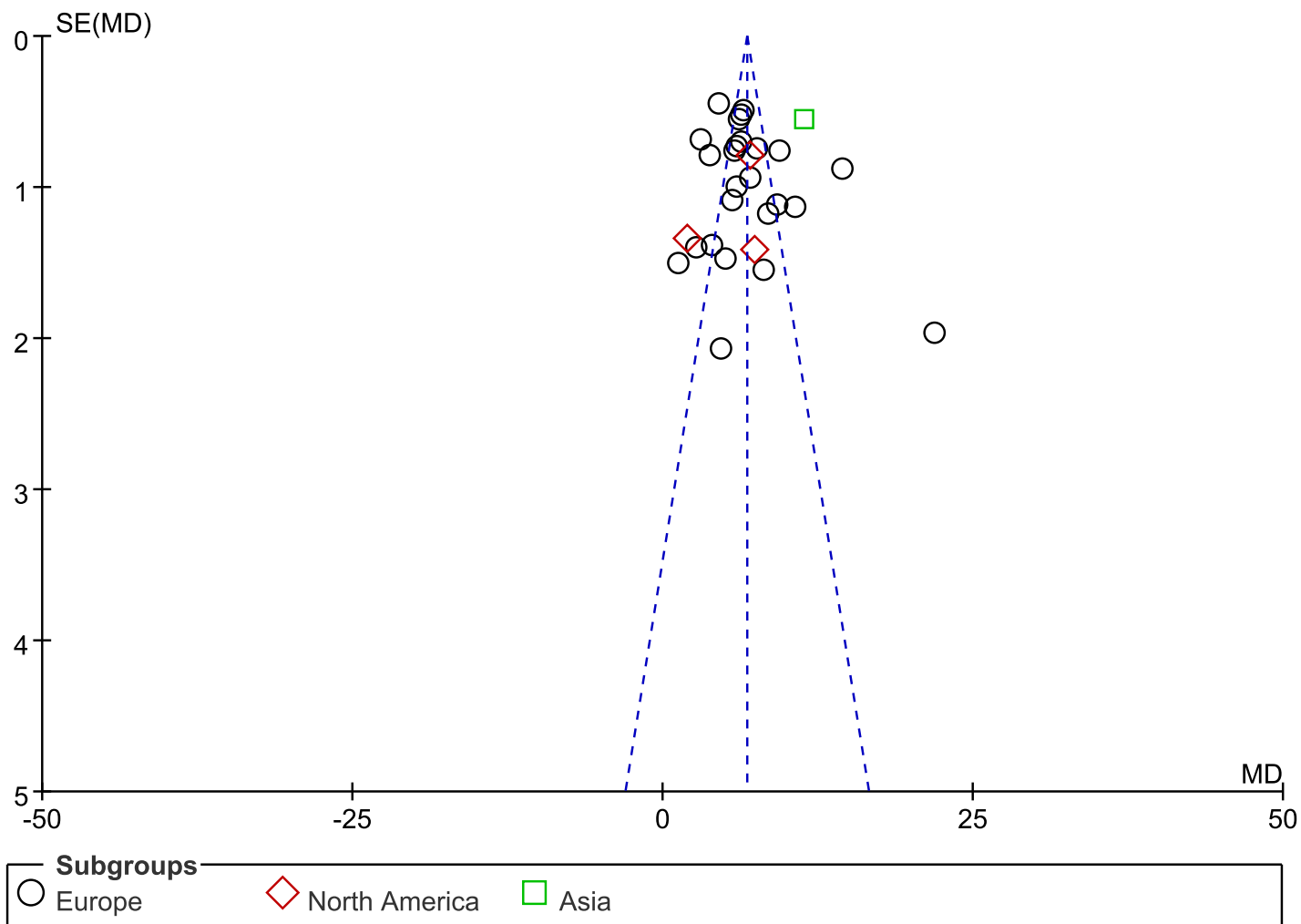

Supplement: Supplemental Information 8 [file peerj-14-21133-s008.pdf]

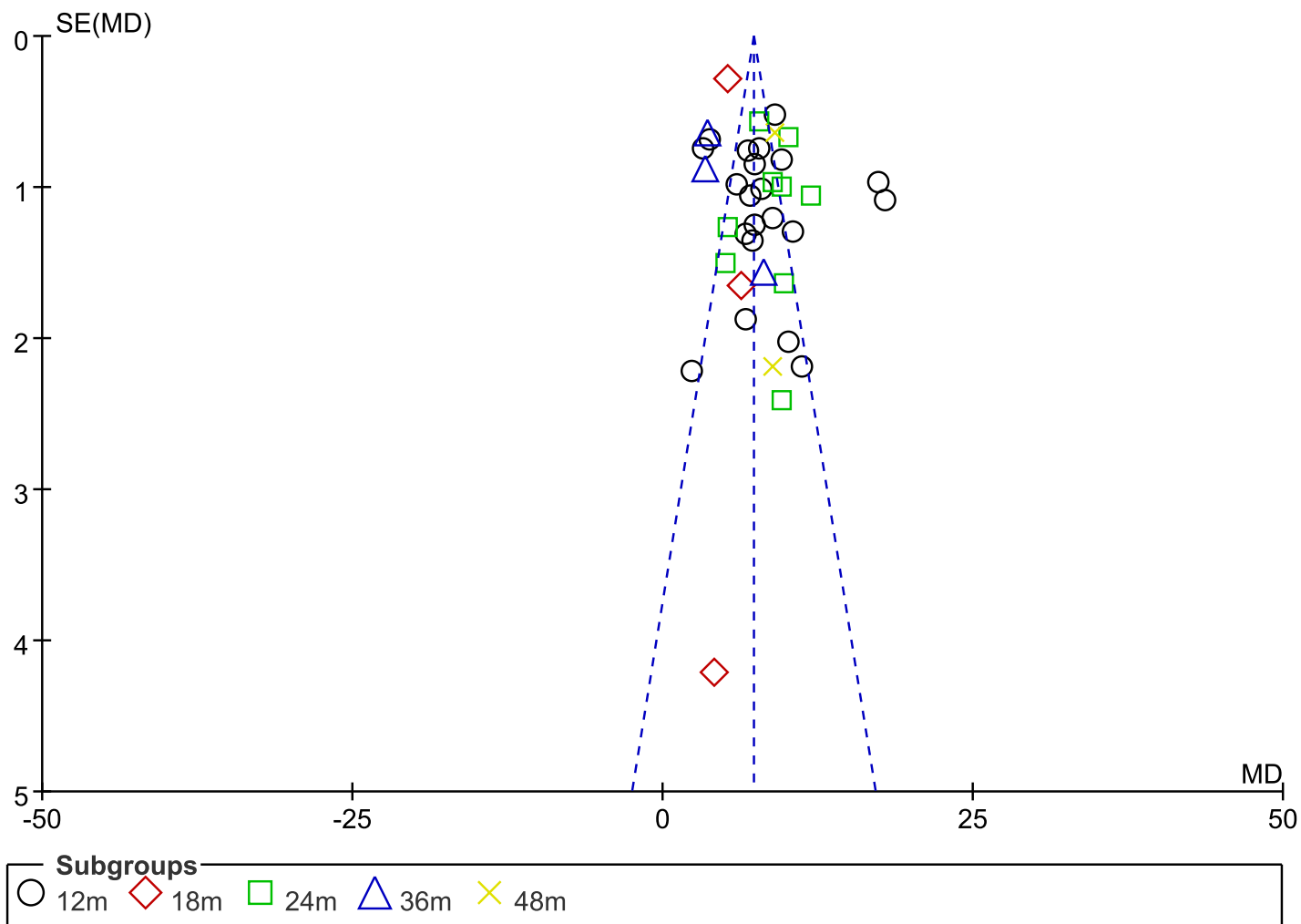

Supplement: Supplemental Information 9 [file peerj-14-21133-s009.pdf]

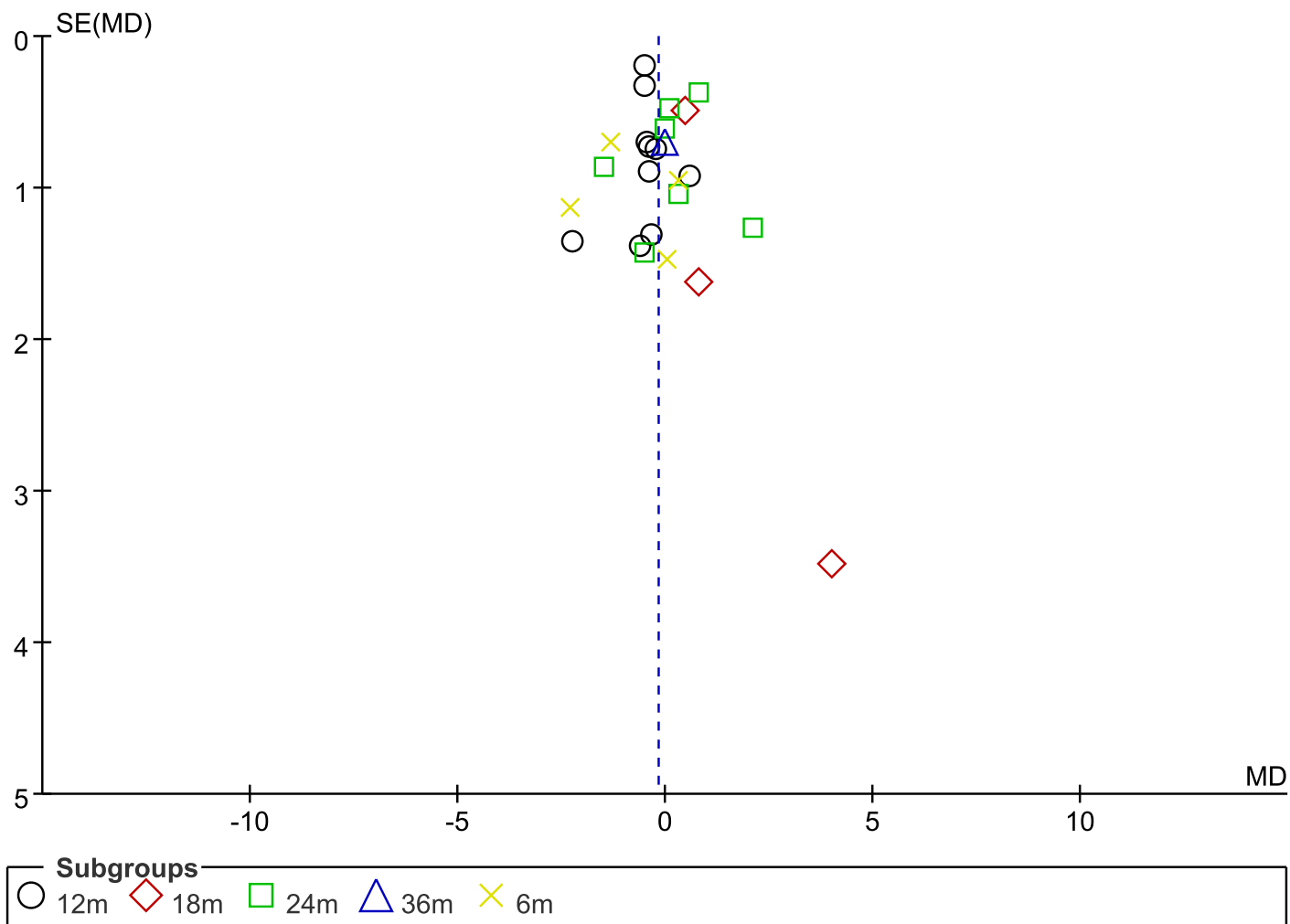

Supplement: Supplemental Information 10 [file peerj-14-21133-s010.pdf]

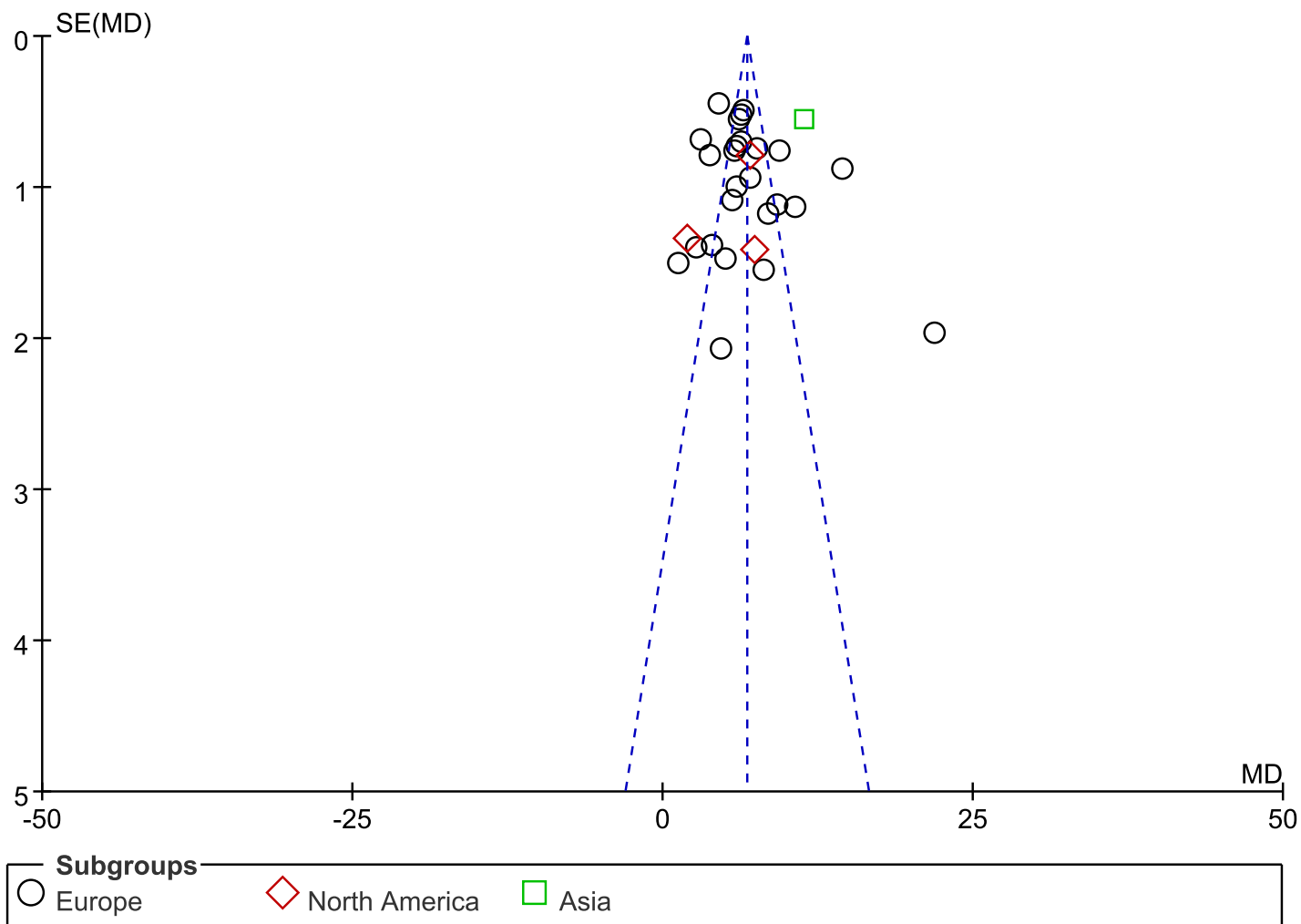

Supplement: Supplemental Information 11 [file peerj-14-21133-s011.pdf]

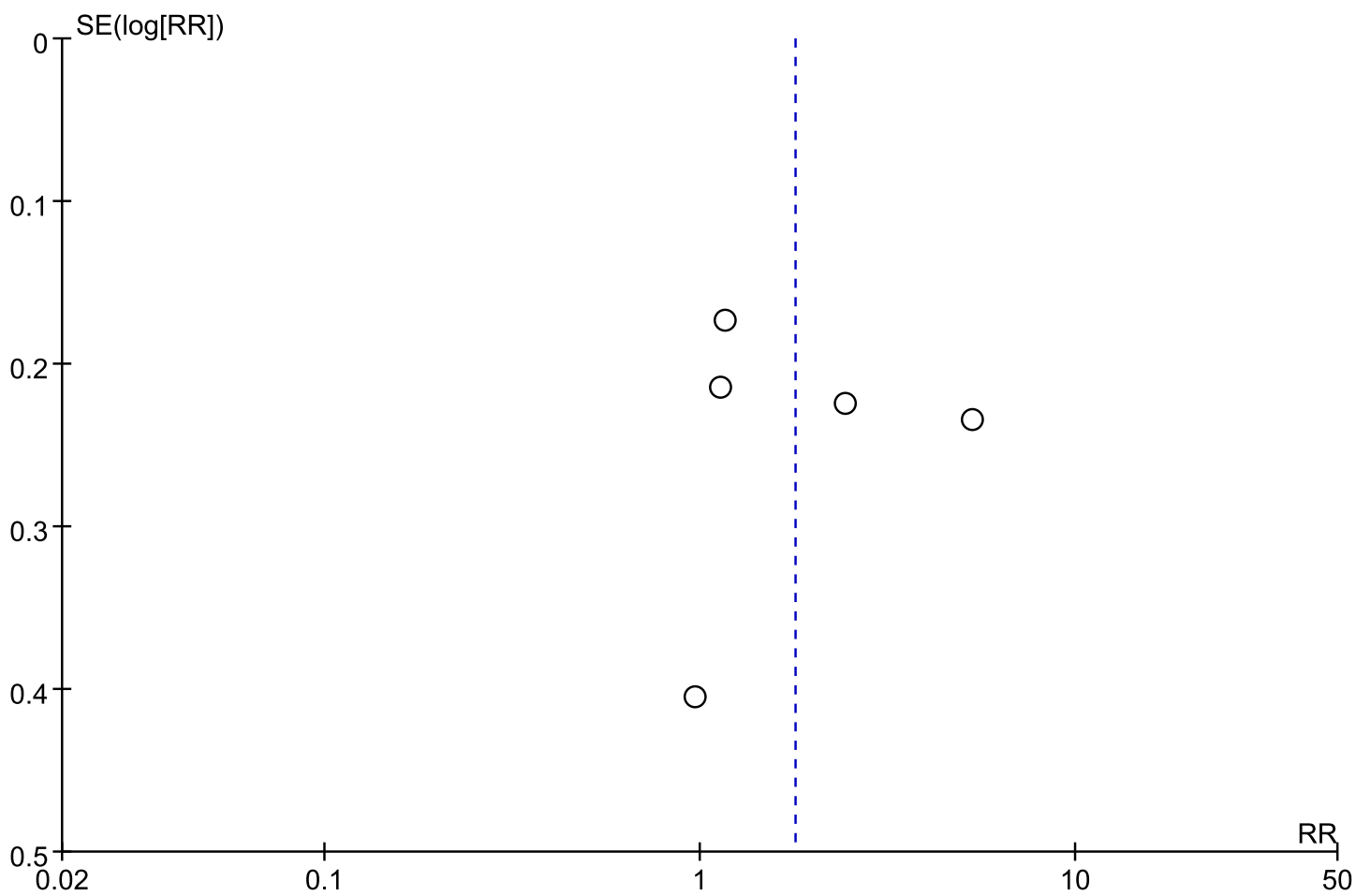

Supplement: Supplemental Information 12 [file peerj-14-21133-s012.pdf]

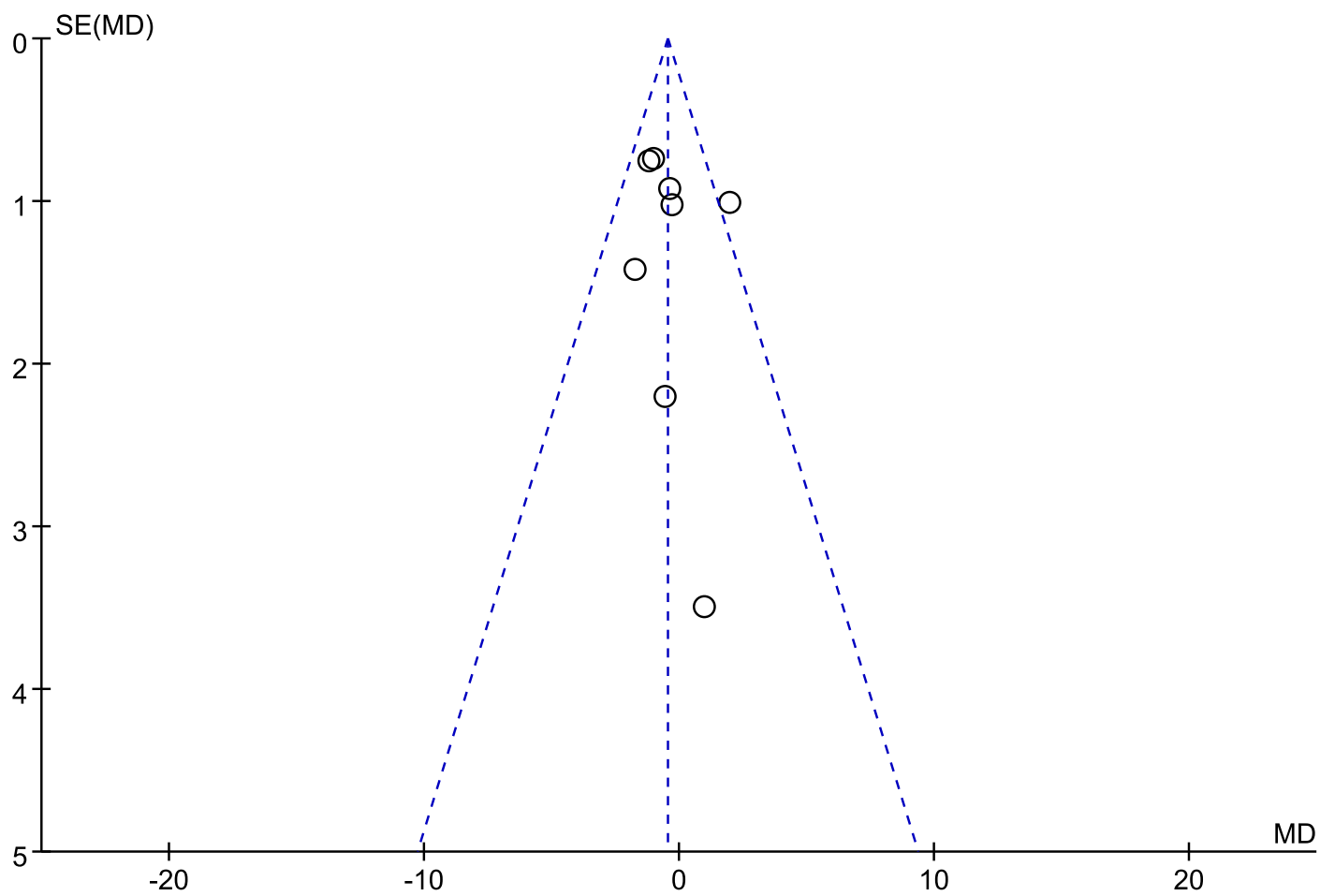

Supplement: Supplemental Information 13 [file peerj-14-21133-s013.pdf]

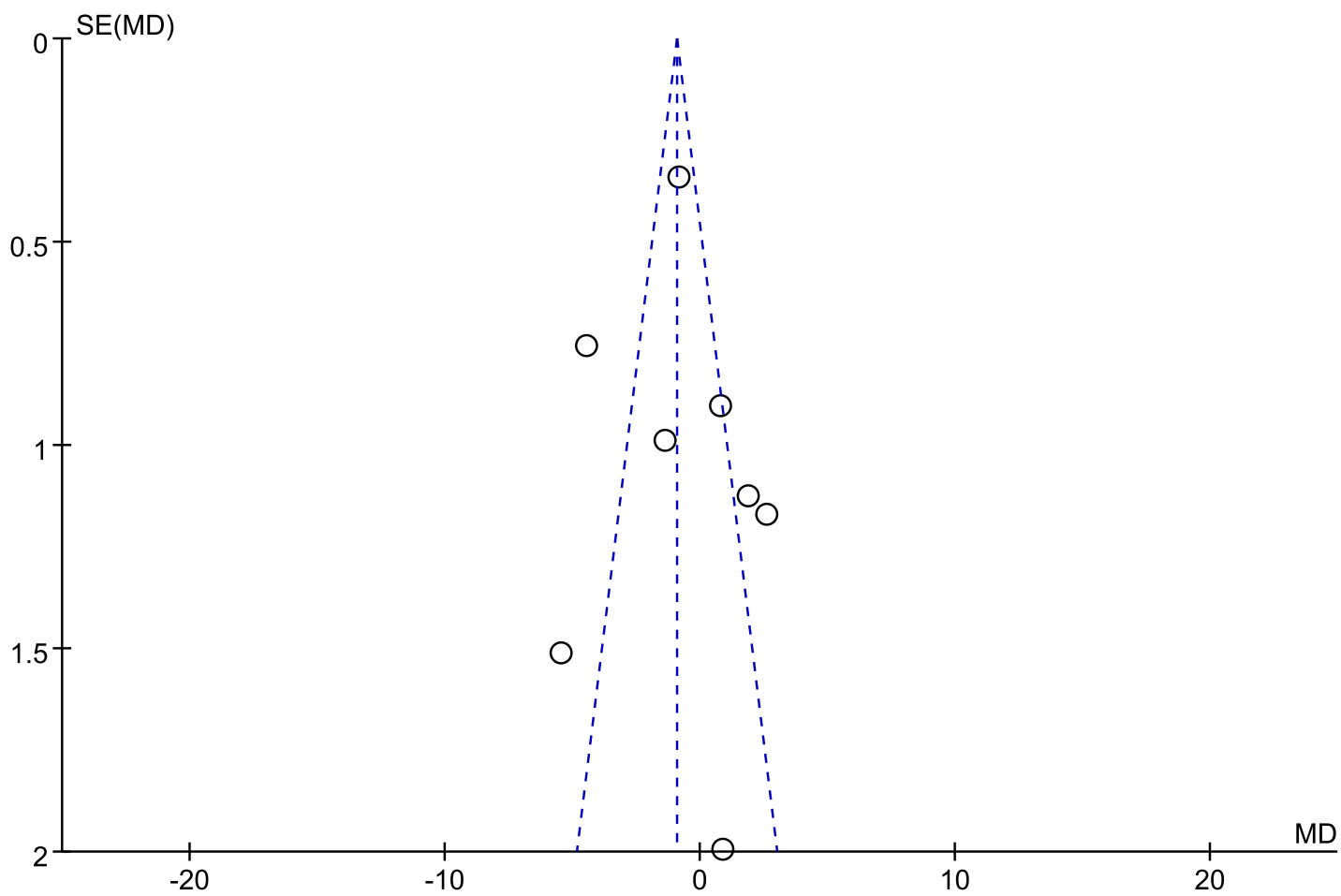

Supplement: Supplemental Information 14 [file peerj-14-21133-s014.pdf]

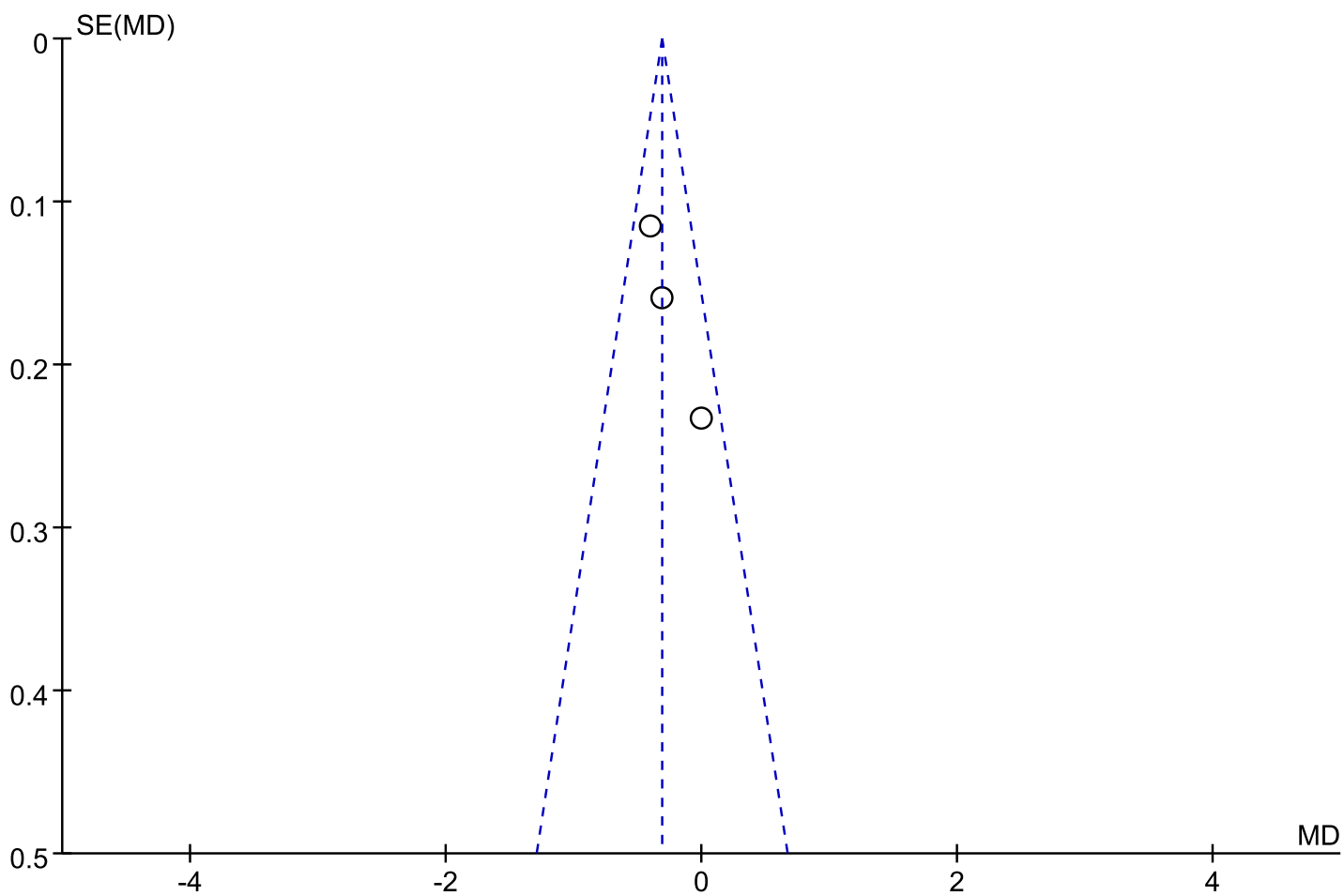

Supplement: Supplemental Information 15 [file peerj-14-21133-s015.pdf]

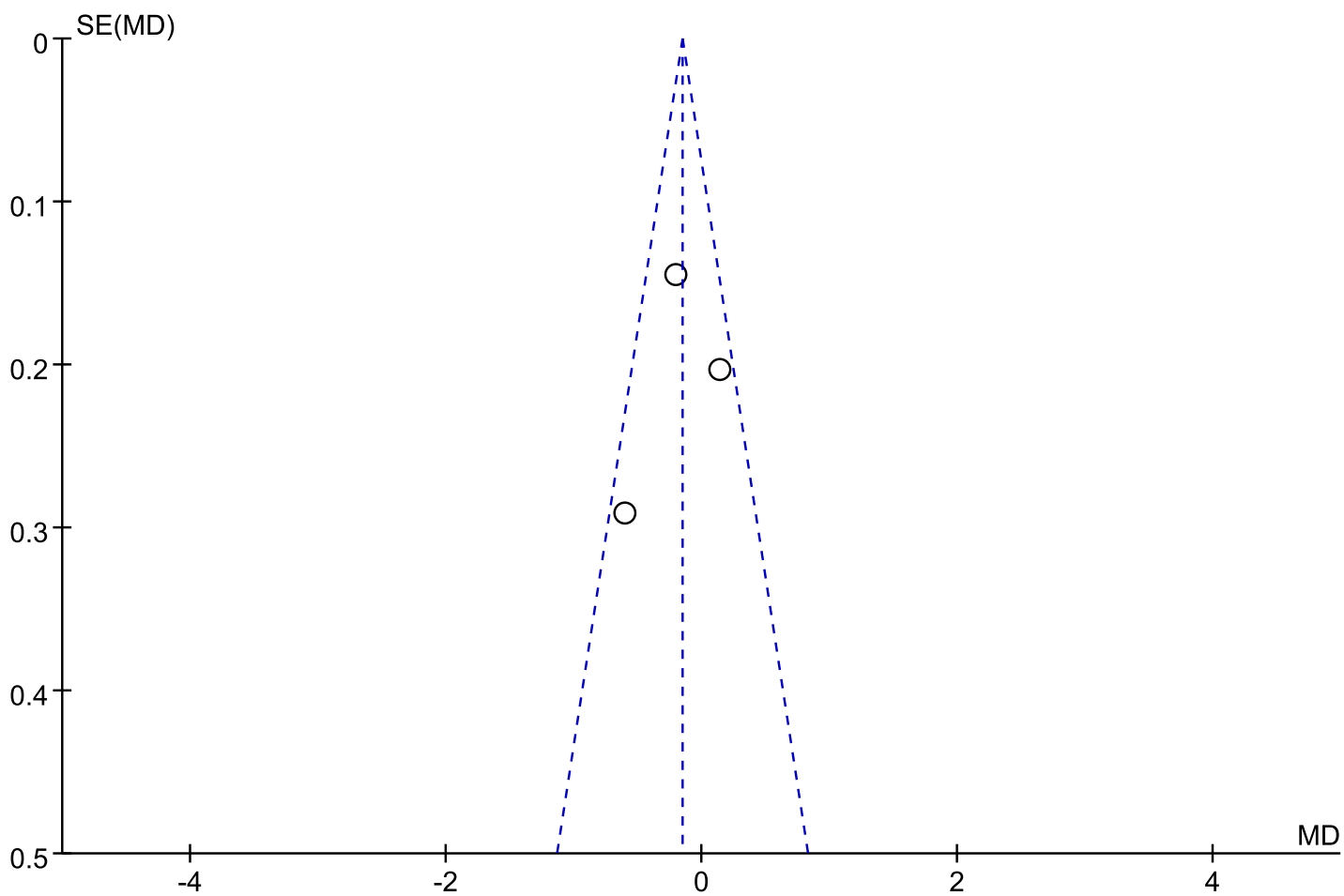

Supplement: Supplemental Information 16 [file peerj-14-21133-s016.pdf]

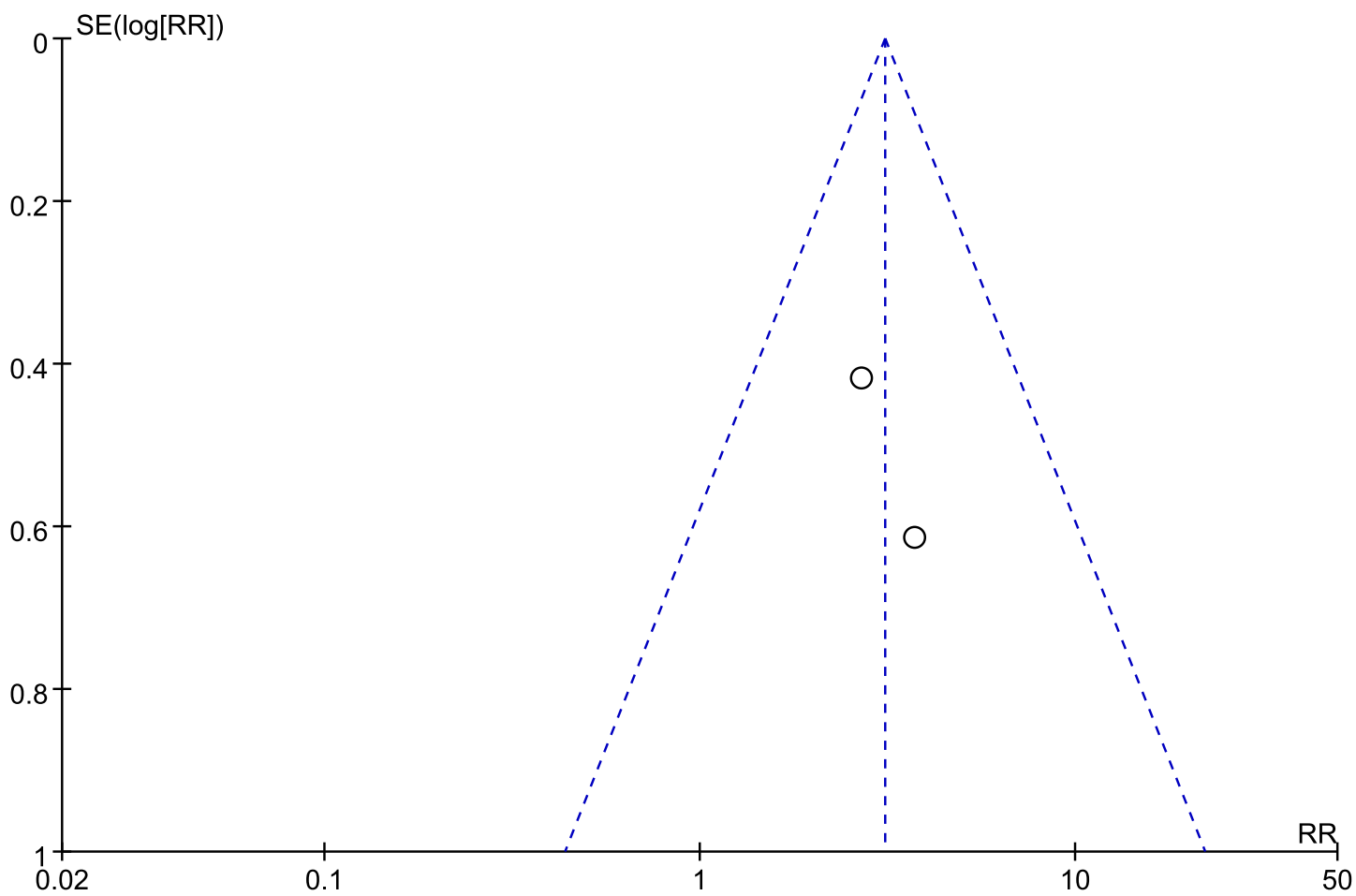

Supplement: Supplemental Information 17 [file peerj-14-21133-s017.pdf]

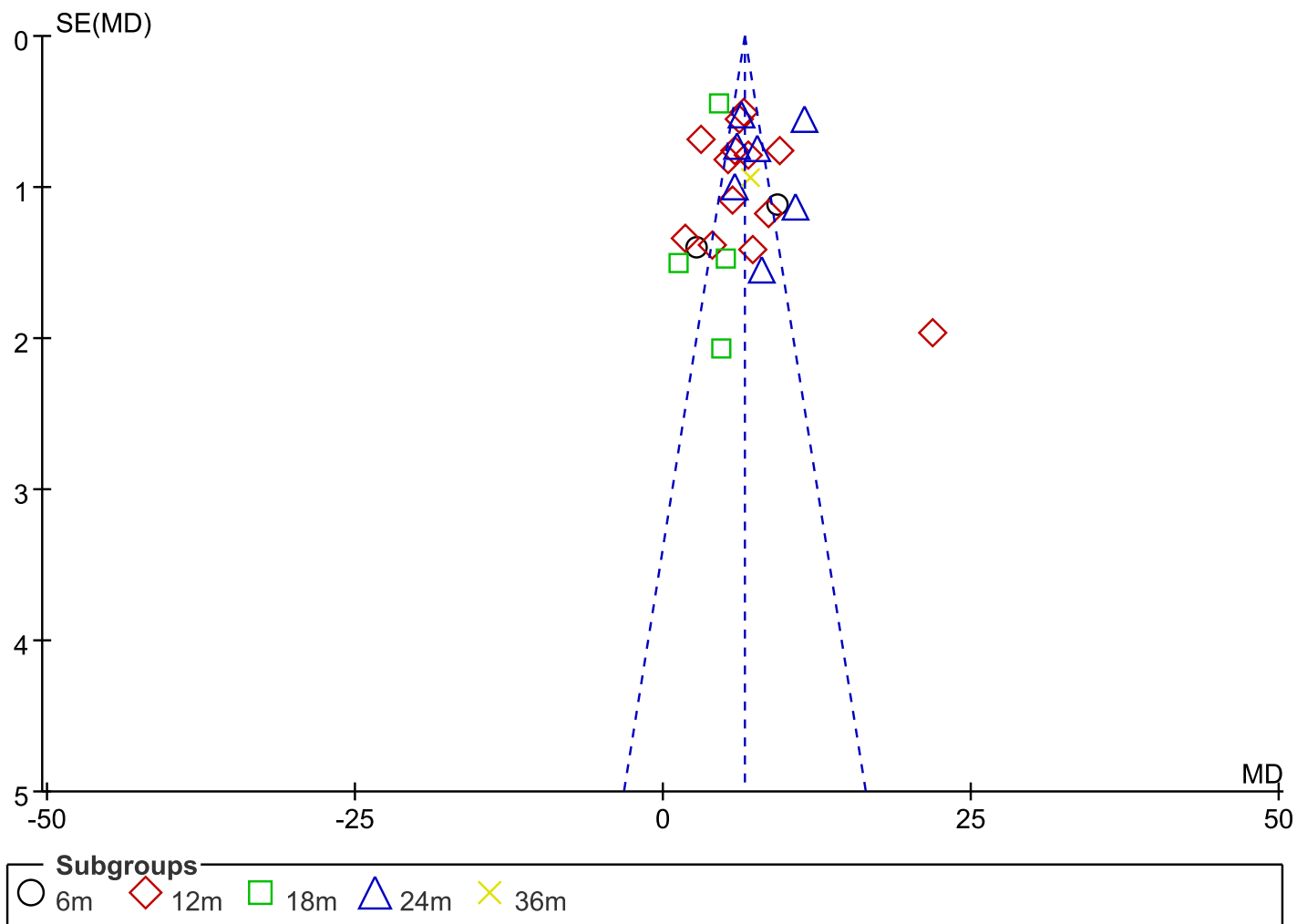

Supplement: Supplemental Information 18 [file peerj-14-21133-s018.pdf]

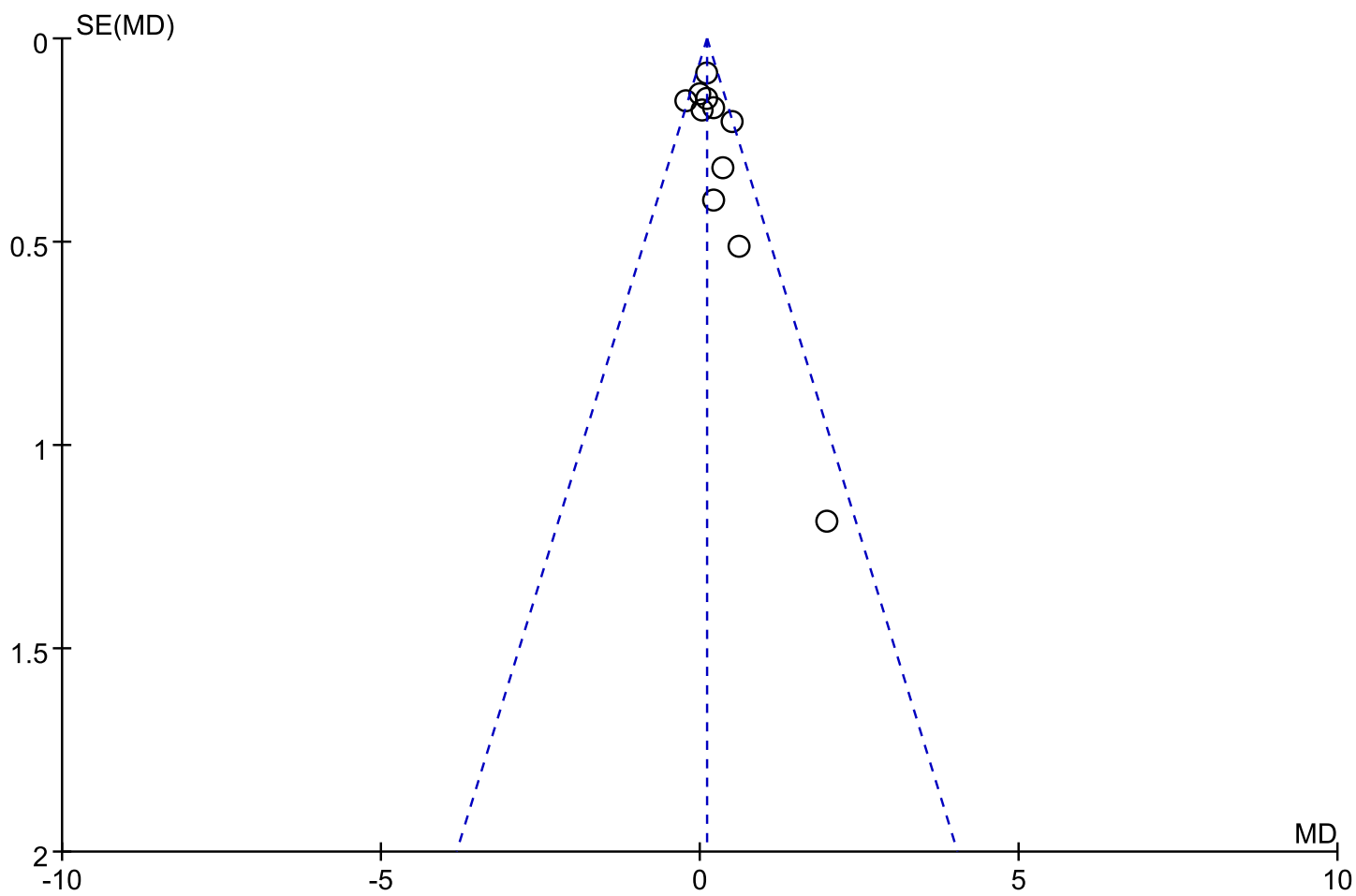

Supplement: Supplemental Information 19 [file peerj-14-21133-s019.pdf]

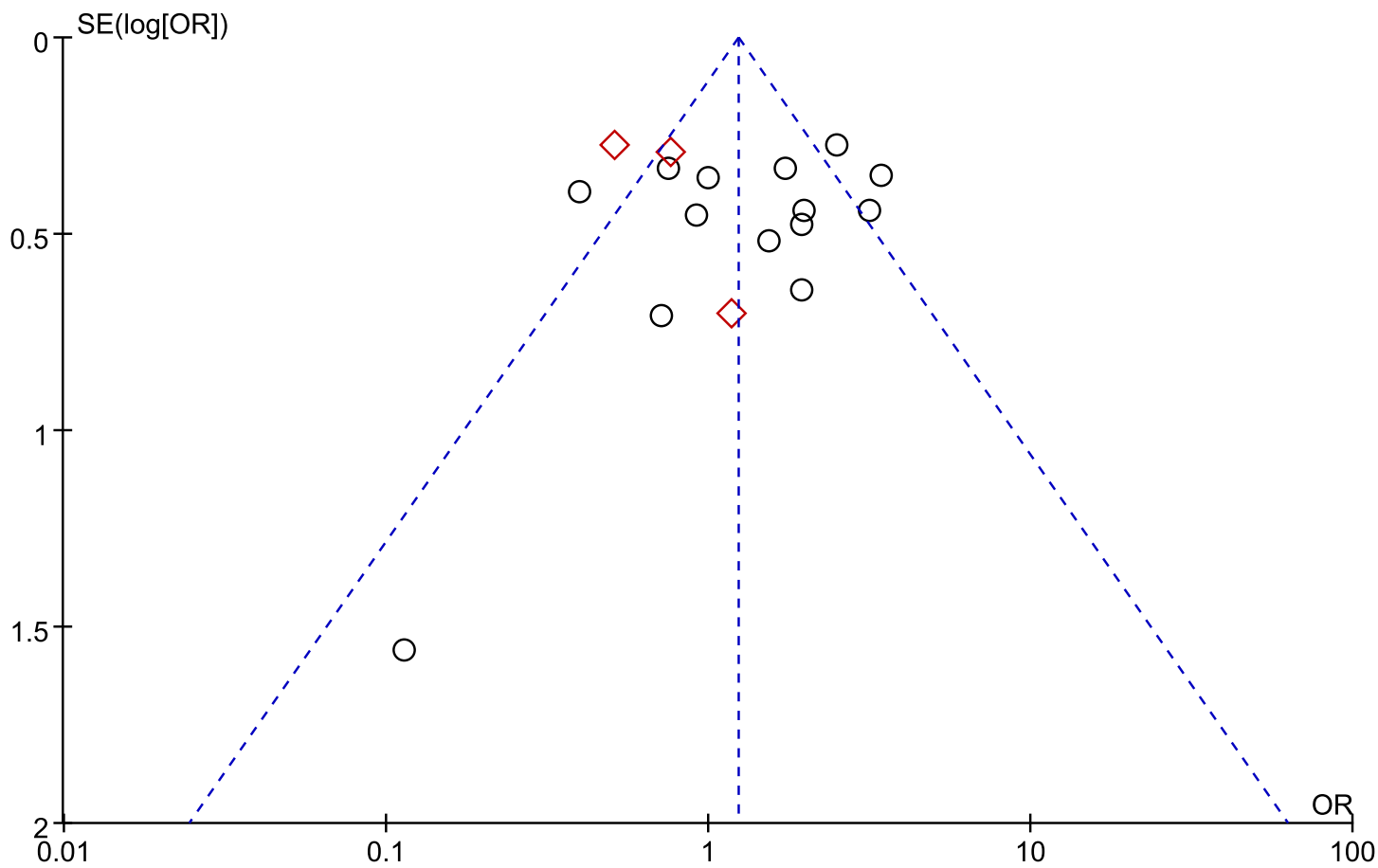

**Subgroups**

○ Xen vs phaco\_Xen    ◇ Xen vs TB

Supplement: Supplemental Information 20 [file peerj-14-21133-s020.pdf]

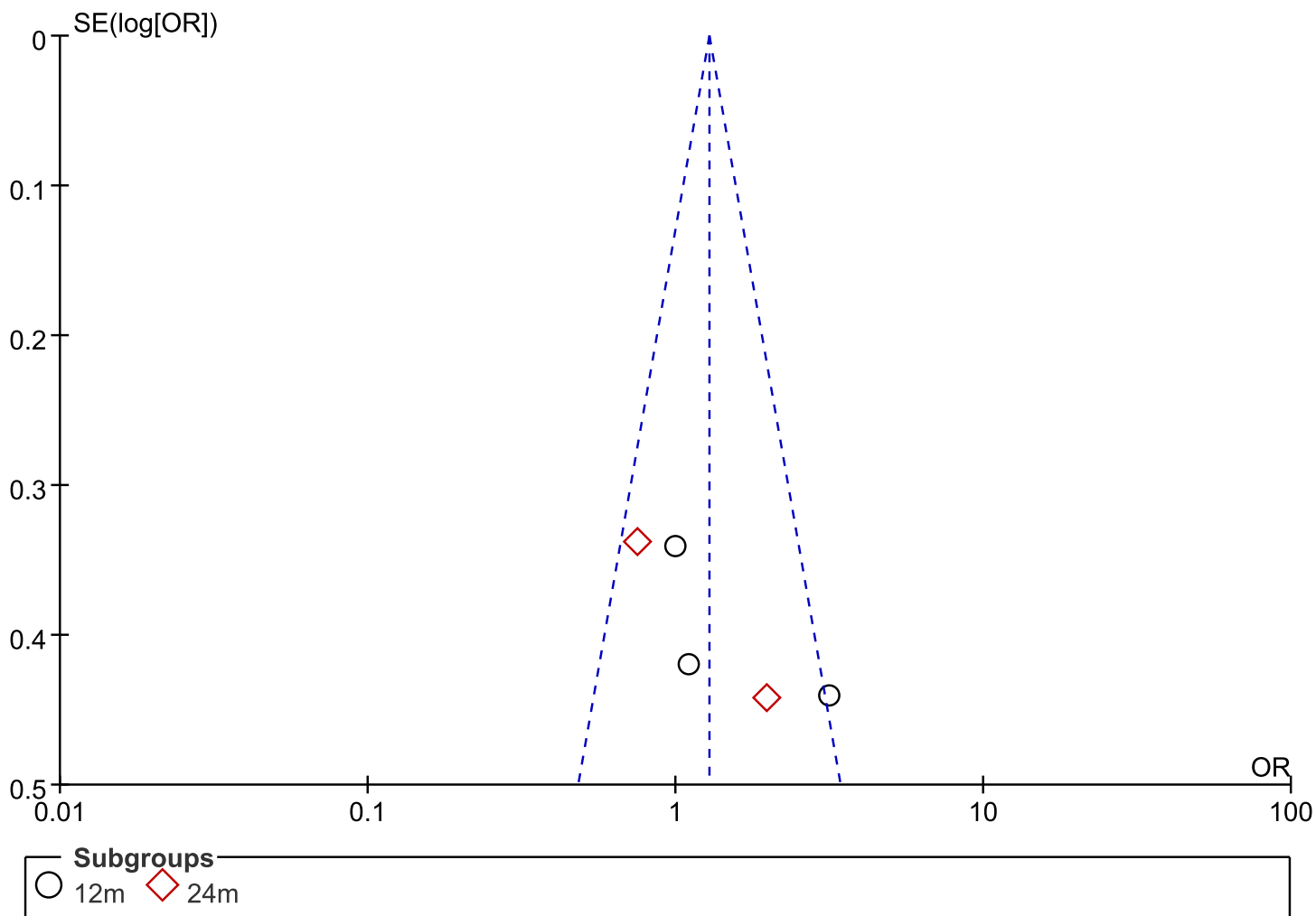

Supplement: Supplemental Information 21 [file peerj-14-21133-s021.pdf]

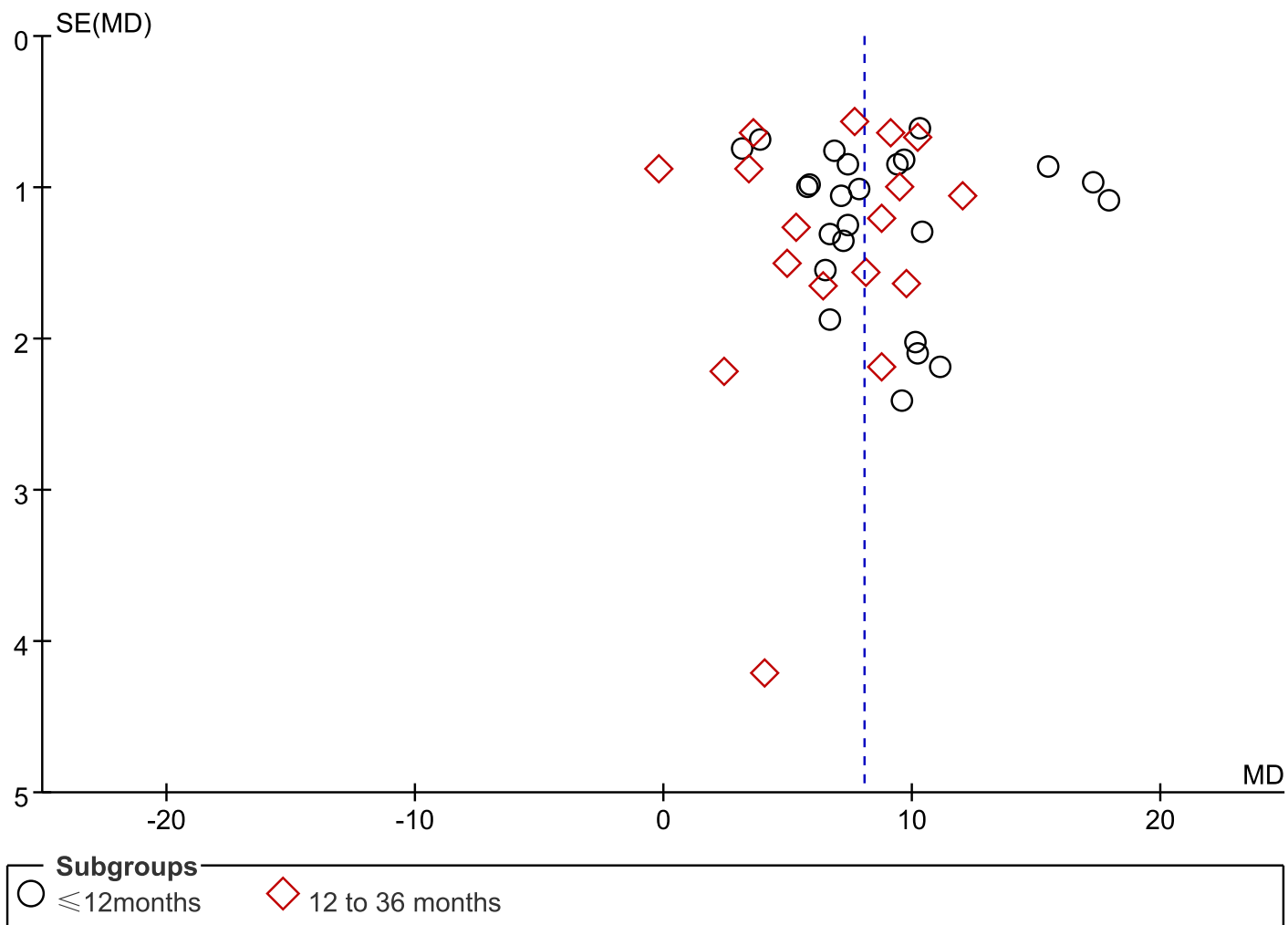

Supplement: Supplemental Information 22 [file peerj-14-21133-s022.pdf]

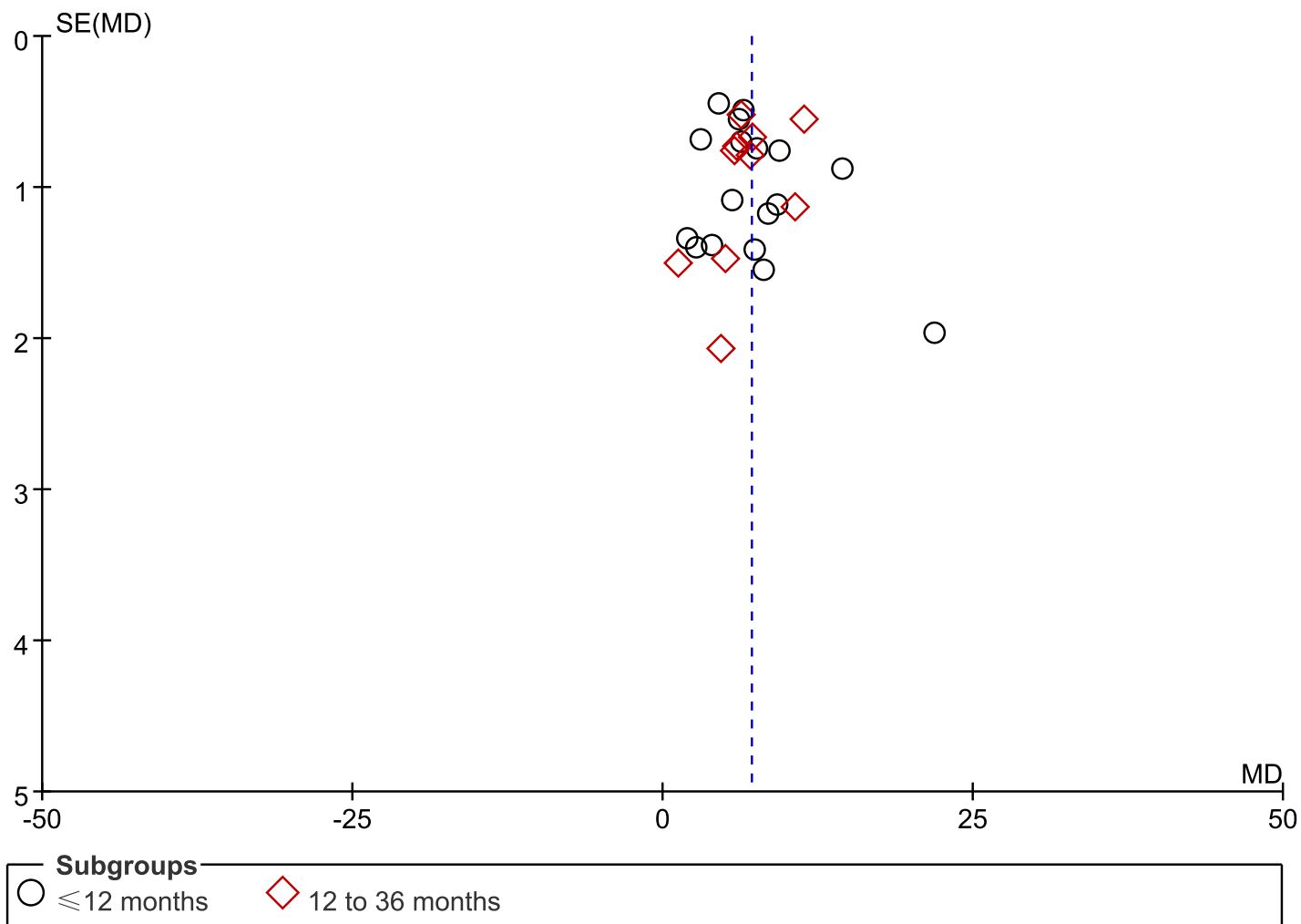

Supplement: Supplemental Information 23 [file peerj-14-21133-s023.pdf]

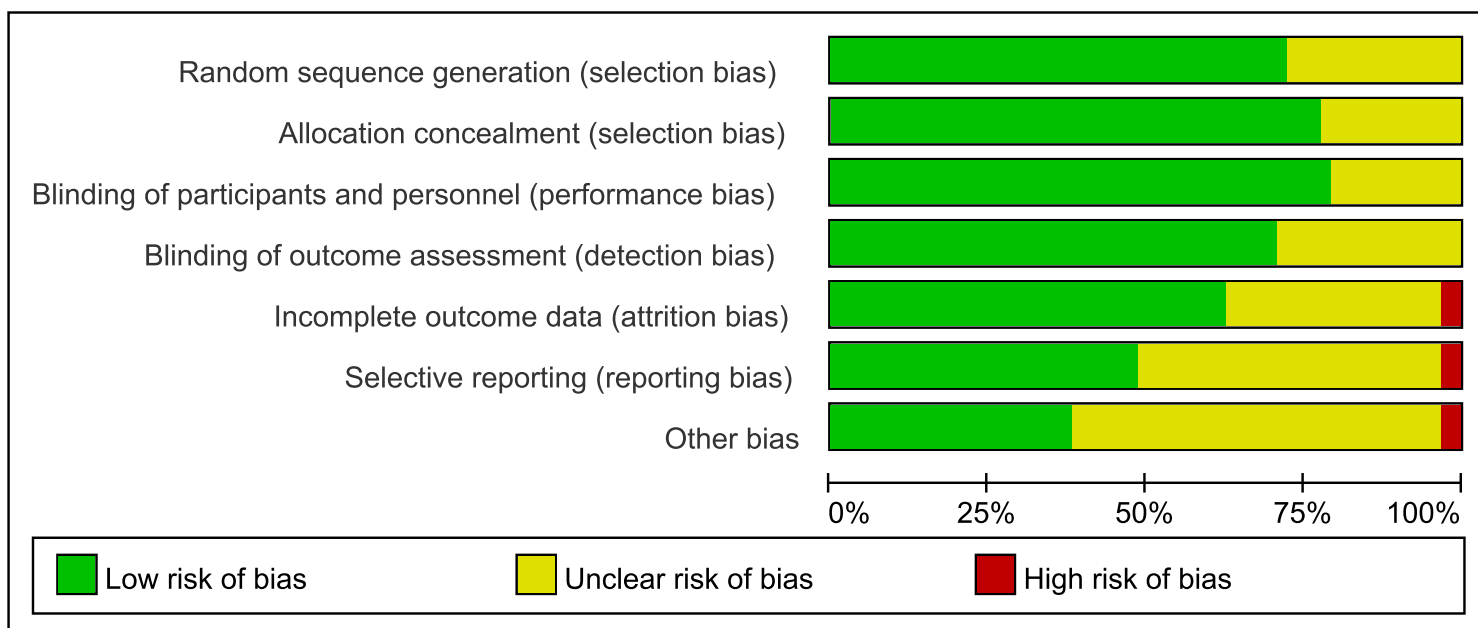

Supplement: Supplemental Information 25 [file peerj-14-21133-s025.pdf]

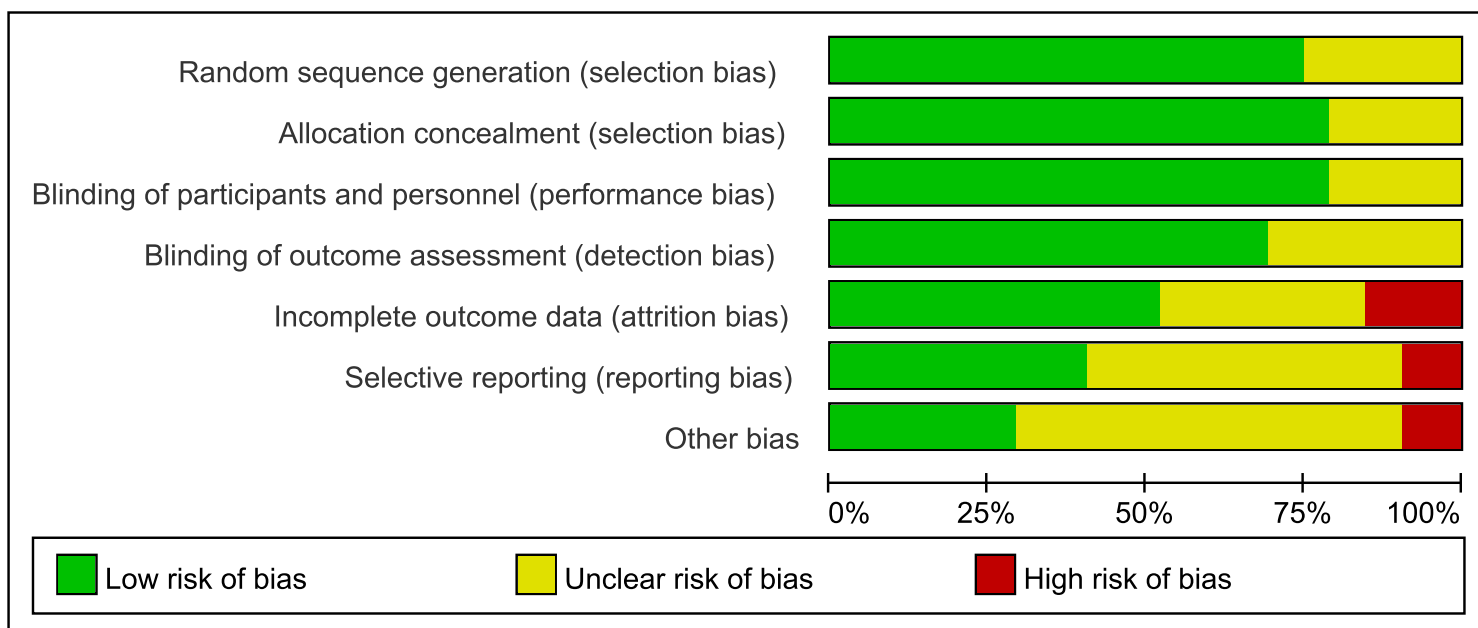

Supplement: Supplemental Information 27 [file peerj-14-21133-s027.zip › raw data_109292_staffFile_SLS_EO/bias1.pdf]
