# Supplementary material for: Comparison of XEN gel stent for management of open-angle glaucoma: a systematic review and meta-analysis
Source: PeerJ. 2026 Jun 9;14:e21133. doi: 10.7717/peerj.21133 (PMC13262562; doi:10.7717/peerj.21133)
Supplement: Supplemental Information 24 [file peerj-14-21133-s024.pdf]

	Random sequence generation (selection bias)	Allocation concealment (selection bias)	Blinding of participants and personnel (performance bias)	Blinding of outcome assessment (detection bias)	Incomplete outcome data (attrition bias)	Selective reporting (reporting bias)	Other bias
Barão, R.C 2020	?	+	+	+	+	+	?
Bufault, J 2020	+	?	?	+	+	+	?
Busch Tobias 2023	?	?	?	?	+	+	?
Cappelli.F 2021	+	+	+	+	?	?	+
Chao, Y.J 2021	+	+	+	+	+	+	?
De Gregorio,A 2018	+	+	+	+	+	?	+
Fea, A. M 2017	?	+	+	?	+	?	?
Fea, A. M 2020	+	+	?	?	?	?	?
Fernández_fGarcía, A 2020（1）	+	+	+	?	+	?	?
FernándezGarcía, A 2020（2）	+	+	+	+	?	+	+
Galal, A 2017	+	+	+	?	−	?	?
Gillmann, K 2020(1)	+	+	+	?	?	?	−
Gillmann, K 2020(2)	+	+	+	+	+	+	+
Gregorio 2017	+	+	+	+	+	+	?
Hengerer, F. H 2018	+	+	+	?	?	?	?
Hengerer, F. H 2019	+	?	+	?	+	+	?
Hohberger, B 2018	?	+	+	+	+	−	?
Ibáñez-Muñoz, A 2020	?	+	+	+	?	?	?
Kalina, AG 2019	+	?	+	?	+	+	+
Karimi, A 2018	+	+	?	?	?	?	?
Laborda__Guirao, T 2020	+	+	+	?	+	+	?
Laroche, D 2019	+	+	+	+	?	?	+
Lavin-Dapena,C 2020	?	+	+	+	−	?	?
Lenzhofer,M 2019（1）	+	+	?	+	+	?	+
Lenzhofer, M 2019（2）	+	+	?	+	+	+	?
Lenzhofer, M 2019（3）	?	+	+	+	+	+	+
Louis Arnould 2024	+	+	+	+	+	+	+
Mansouri, K 2018 a	+	+	+	+	?	?	?
Mansouri, K 2019	+	+	?	+	+	−	?
Marcos Parra, M.T 2019	?	+	+	+	+	?	+
Midha, N 2019	+	+	?	?	+	+	+
Midha, N 2020	+	+	+	+	?	?	?
Oddone, F 2021	+	?	+	+	?	+	+
Olate-Pérez, Á. 2017	?	?	+	?	+	?	+
Olgun, A 2020（1）	+	?	+	?	+	+	?
Olgun, A 2020（2）	?	+	+	+	?	?	?
Papazoglou Anthia 2024	?	?	+	+	+	+	+
Pérez-Torre_grosa, V. T 2016	+	+	+	+	?	?	?
Pirani Vittorio 2024	?	+	+	+	+	+	+
Post, M 2020	+	?	+	+	+	?	?
Rather, P.A. 2020	?	+	+	+	?	?	?
Rauchegger Teresa 2024	+	+	?	?	+	?	?
Reitsamer, H 2019	+	+	+	+	+	+	+
Reitsamer, H 2021	+	+	+	+	+	+	+
Sacch.M 2020	?	+	+	+	?	+	+
Schargus, M 2020	+	+	+	+	+	?	+
Scheres, M. J 2020	+	?	?	+	+	+	+
Sheybani, A 2015	+	+	+	+	?	?	?
Subaşı.S 2021	+	+	+	+	+	?	?
Tan, N.E 2021	+	+	?	+	?	?	?
Tan, S. Z 2018	+	+	+	+	+	+	?
Teus, M. A 2019	+	?	+	+	?	?	?
Theilig.T 2020	+	+	+	+	+	+	+
Torbey Julien 2023	+	+	?	+	+	+	+
Urcola, A 2021	+	+	+	?	?	+	−
Wałek, E 2020	?	+	+	+	+	+	?
Widder, R. A 2018	?	?	+	+	+	+	?
Widder, R. A 2020	+	?	+	?	?	?	?
