# Supplementary material for: Comparison of XEN gel stent for management of open-angle glaucoma: a systematic review and meta-analysis
Source: PeerJ. 2026 Jun 9;14:e21133. doi: 10.7717/peerj.21133 (PMC13262562; doi:10.7717/peerj.21133)
Supplement: Supplemental Information 27 [file peerj-14-21133-s027.zip › raw data_109292_staffFile_SLS_EO/Risk of bias summary.pdf]

Study	Random sequence generation (selection bias)	Allocation concealment (selection bias)	Blinding of participants and personnel (performance bias)	Blinding of outcome assessment (detection bias)	Incomplete outcome data (attrition bias)	Selective reporting (reporting bias)	Other bias
Barão, R.C 2020	?	+	+	+	+	+	?
Bufault, J 2020	+	?	?	+	+	+	?
Cappelli.F 2021	+	+	+	+	?	?	+
Chao, Y.J 2021	+	+	+	+	+	+	?
De Gregorio,A 2018	+	+	+	+	+	?	+
Fea, A. M 2017	?	+	+	?	+	?	?
Fea, A. M 2020	+	+	?	?	−	?	?
Fernández_fGarcía, A 2020（1）	+	+	+	?	+	?	?
FernándezGarcía, A 2020（2）	+	+	+	+	?	+	+
Galal, A 2017	+	+	+	?	−	?	?
Gillmann, K 2020(1)	+	+	+	?	?	?	?
Gillmann, K 2020(2)	+	+	+	?	?	−	?
Gregorio 2017	+	+	?	+	+	?	−
Hengerer, F. H 2018	+	+	+	?	?	?	?
Hengerer, F. H 2019	+	?	+	?	+	+	?
Hohberger, B 2018	?	+	+	+	+	−	?
Ibáñez-Muñoz, A 2020	?	+	+	+	−	?	?
Kalina, AG 2019	+	?	+	?	−	+	?
Karimi, A 2018	+	+	?	?	?	−	?
Laborda__Guirao, T 2020	+	+	+	?	+	+	−
Laroche, D 2019	+	+	+	+	?	−	+
Lavin-Dapena,C 2020	?	+	+	+	−	?	?
Lenzhofer,M 2019（1）	+	+	?	+	+	?	+
Lenzhofer, M 2019（2）	+	+	?	+	+	+	?
Lenzhofer, M 2019（3）	?	+	+	+	+	+	+
Mansouri, K 2018 a	+	+	+	+	?	?	?
Mansouri, K 2019	+	+	?	+	+	−	?
Marcos Parra, M.T 2019	?	+	+	+	+	?	+
Midha, N 2019	+	+	?	?	+	+	+
Midha, N 2020	+	+	+	+	−	?	?
Oddone, F 2021	+	?	+	+	?	+	+
Olate-Pérez, Á. 2017	?	?	?	?	−	?	?
Olgun, A 2020（1）	+	?	+	?	+	+	?
Olgun, A 2020（2）	?	+	+	+	?	?	−
Pérez-Torre_grosa, V. T 2016	+	+	+	+	?	?	?
Post, M 2020	+	?	+	+	+	?	?
Rather, P.A. 2020	?	+	+	+	?	?	?
Reitsamer, H 2019	+	+	+	+	+	+	+
Reitsamer, H 2021	+	+	+	+	+	+	+
Sacch.M 2020	?	+	+	+	?	+	+
Schargus, M 2020	+	+	+	+	+	?	+
Scheres, M. J 2020	+	?	?	+	+	+	+
Sheybani, A 2015	+	+	+	+	?	?	?
Subaşı.S 2021	+	+	+	+	+	?	?
Tan, N.E 2021	+	+	?	+	?	?	?
Tan, S. Z 2018	+	+	+	+	+	+	?
Teus, M. A 2019	+	?	+	+	−	?	−
Theilig.T 2020	+	+	+	+	+	+	+
Urcola, A 2021	+	+	+	?	?	+	−
Wałek, E 2020	?	+	+	+	+	+	?
Widder, R. A 2018	?	?	+	+	+	+	?
Widder, R. A 2020	+	?	+	?	?	?	?
